# Current status of the rapid decline in renal function due to diabetes mellitus and its associated factors: analysis using the National Database of Health Checkups in Japan

**DOI:** 10.1038/s41440-023-01185-2

**Published:** 2023-02-02

**Authors:** Makoto Fujii, Yuko Ohno, Asuka Ikeda, Kayo Godai, Yaya Li, Yuko Nakamura, Daisuke Yabe, Kazuyo Tsushita, Naoki Kashihara, Kei Kamide, Mai Kabayama

**Affiliations:** 1 Division of Health Sciences, Osaka University Graduate School of Medicine, 1-7 Yamadaoka, Suita, Osaka 565-0871 Japan; 2 Department of Diabetes, Endocrinology and Metabolism and Department of Rheumatology and Clinical Immunology, Gifu University Graduate School of Medicine, 1-1 Yanagido, Gifu, Gifu 501-1193 Japan; 3 Graduate Schools of Nutrition Sciences, Kagawa Nutrition University, 3-9-21 Chiyoda, Sakado, Saitama 350-0288 Japan; 4 Department of Nephrology and Hypertension, Kawasaki Medical School, 577 Matsushima, Kurashiki, Okayama 701-0192 Japan

**Keywords:** Diabetic kidney disease, Diabetes mellitus, Diabetic nephropathy, National Database of Health Checkups, Renal function

## Abstract

The increasing number of patients undergoing dialysis due to diabetes mellitus (DM) is causing serious economic problems, and its reduction is an urgent policy issue in developed countries, including Japan. We aimed to assess the association between the annual rapid decline in renal function and health checkup measures, including blood pressure, to identify health guidance targets for preventing diabetic nephropathy (DN) and diabetic kidney disease (DKD) among individuals in a medical checkup system (“Tokuteikenshin” program) in 2018. This longitudinal analysis included 3,673,829 individuals who participated in the “Tokuteikenshin” program in 2018, had hemoglobin A1c (HbA1c) levels ≥5.6%, were available for follow-up, and underwent estimated glomerular filtration rate (eGFR) evaluation. We estimated the incidence of the relative annual decrease in eGFR ≥10% per 1000 person-years and odds ratios to evaluate the rapid decline in renal function and determine health guidance goals and their role in preventing DN and DKD. Overall, 20.83% of patients with DM had a rapid decline in renal function within the observation period. A rapid decline in renal function was associated with high systolic blood pressure, poor or strict DM control, increased urinary protein excretion, and decreased blood hemoglobin levels. The incidence of rapid decline in renal function is higher in DM, and appropriate systolic blood pressure and glycemic control are important to prevent the progression to DN or DKD. Our findings will be useful for researchers, clinicians, and other public health care members in establishing effective health guidance and guidelines for CKD prevention.

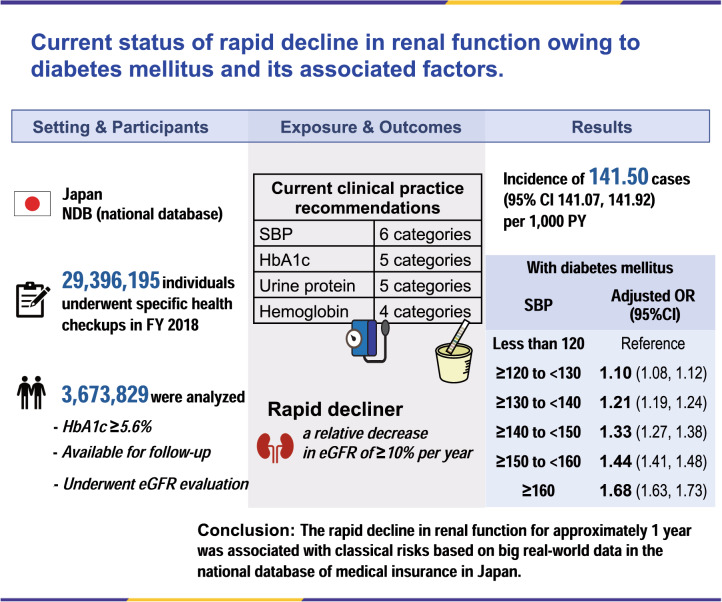

## Introduction

Diabetic nephropathy (DN) is classically diagnosed based on the development of sustained proteinuria, followed by gradual decreases in the estimated glomerular filtration rate (eGFR) [1]. However, several studies have recently reported a trajectory of renal function (i.e., fast renal decline in eGFR without proteinuria) different from this classic phenotype [2, 3]. Recently, diabetic kidney disease (DKD), a more comprehensive concept than DN, has been proposed that includes typical diabetic nephropathy and atypical diabetes-related renal disease with decreased GFR without overt albuminuria. Furthermore, diabetes-associated chronic kidney disease (CKD) is a broader concept that includes patients with renal disease not directly related to diabetes (e.g., immunoglobulin A nephropathy, polycystic kidney disease) who have diabetes mellitus [4]. Renal damage due to diabetes mellitus (DM) is considered one of the main causes of CKD, and the number of patients with CKD in Japan is estimated to be 13.3 million [5]. The proportion of CKD attributable to DM has continued to increase and has been the leading cause of dialysis in Japan since 1998 [6]. The increasing number of patients undergoing dialysis is causing serious economic problems, and its reduction is an urgent policy issue in developed countries, including Japan.

Japan has universal public health care insurance coverage that has allowed affordable access to outpatient, inpatient, and pharmaceutical care since 1961 [7]. For medical insurers (health care insurance system), health checkups and health guidance focusing on visceral fat obesity based on specific health checkup programs are required for insured people and dependents aged ≥40 years. This medical checkup system is known as the “Tokuteikenshin” program. The Ministry of Health, Labor, and Welfare (MHLW) in Japan has all the data on participants in the “Tokuteikenshin” program in the National Health Insurance Claims and Specific Health Checkups of Japan Database (NDB) [8]. Although evidence exists on preventing nephropathy among patients with DM, there is insufficient evidence among the large number of individuals in the “Tokuteikenshin” program or on those who discontinue treatment to address this issue among medical institution patients alone [9]. Additionally, comparisons are needed for patients with or without DM. However, most previous studies were based on medical records of patients with DM attending outpatient clinics or hospitals, and few studies have been conducted using nationwide population-based data, including those of individuals registered in the “Tokuteikenshin” program [3].

Therefore, we aimed to clarify the incidence of rapid decline in renal function based on comprehensive information from the NDB, including those of individuals in the “Tokuteikenshin” program. Furthermore, we aimed to investigate the factors associated with the rapid decline in renal function and establish effective health guidance to prevent an increase in the number of people on dialysis caused by DM.

Point of view

- Clinical relevance
  This study aligns the management goal of hypertension among Asian diabetic patients.
- Future direction
  To determine the management goal for elderly diabetic patients in addition to pharmacotherapy in Asia.
- Considerations for the Asian population
  Patients with comorbidities.
  Patients who do not undergo specific medical examinations.
  Drug treatment, including cardiovascular risk and long-term effects in Asia.

## Methods

### Study design

We conducted a nationwide population-based cohort study.

### Data source and data extraction

We obtained and analyzed anonymized nationwide data on participants who underwent a specific health checkup between fiscal year (FY) 2018 and FY2019 by the MHLW in Japan. For anonymization, we used a unique identification number (id1n) generated based on health insurance code, birthdate, and sex and included in the NDB. Data were extracted by a company unrelated to the researchers that was commissioned by the MHLW.

### Participants

**Fig. 1:**
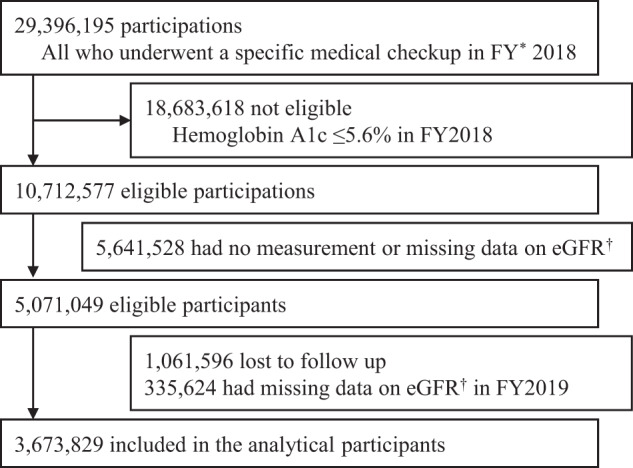
Flowchart of the study participants

In FY2018, 29,396,195 individuals underwent specific health checkups. Of these, those with a hemoglobin A1c (HbA1c) level ≥5.6% (10,712,577 participants) were eligible for renal function tests. Overall, 3,673,829 participants were included in the final analysis; those whose eGFR could not be assessed at baseline and those who did not have a specific health checkup in FY2019 were excluded, as shown in Fig. 1.

### Outcome measures

The main outcomes of interest in this study were the incidence of a relative decrease in eGFR of ≥10% per 1000 person-years (PY) and the evaluation of the relationship between the rapid decline in renal function and management goals for DM or chronic renal failure. Incidence rate ratios (IRRs) and odds ratios (ORs) were used to evaluate the rapid decline in renal function and clinical management goals.

### Definition of rapid decline in renal function and diagnostic criteria for DM

The rate of eGFR decline was determined in units of “mL/min/1.73 m^2^/year” (rate defined as the “slope”) and “%/year” (rate defined as the “%slope”) based on a previous study [10]. The following formula was used to evaluate renal function over time using the eGFR values in the two measurements. The components of the equations included the eGFR value in FY2018 or FY2019 and PY.

$${{{{{{{\mathrm{slope}}}}}}}}\,{{{{{{{\mathrm{in}}}}}}}}\,{{{{{{{\mathrm{eGFR = eGFR}}}}}}}}_{{{{{{{{\mathrm{FY2018}}}}}}}}} - {{{{{{{\mathrm{eGFR}}}}}}}}_{{{{{{{{\mathrm{FY2019}}}}}}}}}/{{{{{{{\mathrm{PY}}}}}}}}$$

$${{{{{{{\mathrm{\% slope}}}}}}}}\,{{{{{{{\mathrm{in}}}}}}}}\,{{{{{{{\mathrm{eGFR = }}}}}}}}\frac{{{{{{{{{\mathrm{eGFR}}}}}}}}_{{{{{{{{\mathrm{FY2018}}}}}}}}} - {{{{{{{\mathrm{eGFR}}}}}}}}_{{{{{{{{\mathrm{FY2019}}}}}}}}}}}{{{{{{{{{\mathrm{eGFR}}}}}}}}_{{{{{{{{\mathrm{FY2018}}}}}}}}}}}{{{{{{{\mathrm{ \times 100}}}}}}}}/{{{{{{{\mathrm{PY}}}}}}}}$$

The normal eGFR decline was 1 mL/min/1.73 m^2^/year after the age of 40 years in previous studies [11, 12]. The slope of eGFR changes over time varied widely from −72 to −3.0 mL/min/1.73 m^2^/year among individuals and was reflected as a very fast, fast, moderate, or slow decline [13–18]. In this study, a rapid rate of eGFR change during the first year of the trial was defined as a −10%/year decline [4, 19]. An eGFR of <60 was defined as CKD. The classification of DM was based on an HbA1c level ≥6.5%, fasting plasma glucose (FPG) level of 126 mg/dL, casual plasma glucose (CPG) level of ≥200 mg/dL, or those who self-reported their use of antidiabetes medication in the “Tokuteikenshin” program [20].

### Medical checkup data

The medical checkup data covered the entire population of Japan, and data were extracted from the NDB using a similar methodological framework as reported previously [8]. Briefly, the medical checkup data were extracted by the MHLW without the involvement of the researchers. The extracted baseline data included sociodemographic variables such as age and sex; basic variables such as height, weight, BMI (body mass index), SBP (systolic blood pressure), and diastolic blood pressure; laboratory test results such as eGFR, triglycerides, high-density lipoprotein cholesterol (HDL-C), low-density lipoprotein cholesterol (LDL-C), aspartate aminotransferase, alanine aminotransferase, gamma-glutamyl transferase, FPG, CPG, HbA1c, hemoglobin (Hb), red blood cell count, urine protein, and urine glucose levels; medications such as antihypertensive drugs, oral antidiabetes drugs, and lipid-lowering drugs; medical history variables such as stroke, heart disease, kidney disease, and anemia; and lifestyle factors such as the amount of alcohol consumed and smoking status.

Cutoff values for demographic indicators, blood tests, and lifestyle were set based on the management goals for each indicator in Japanese guidelines [4, 10, 19, 20]. Specifically, BMI was divided into three categories: <18.5, ≥18.5 to <25, and ≥25; SBP was divided into six categories: <120, ≥120 to <130, ≥130 to <140, ≥140 to <150, ≥150 to <160, and ≥160 mmHg. During the study period, for HbA1c measurement, the National Glycohemoglobin Standardization Program was used. HbA1c was divided into six categories: <6.2%, ≥6.2 to <6.9, ≥6.9 to <7.4, ≥7.4 to <8.4, and ≥8.4; FPG was divided into four categories: <110, ≥110 to ≥130, ≥130 to ≥160, and ≥160 mg/dL. LDL-C levels were divided into three categories: <100, ≥100 to <120, and ≥120 mg/dL. Hb level was divided into four categories: 9, ≥9 to <11, ≥11 to <13, and ≥ 13g/dL.

### Statistical analysis

The baseline and clinical characteristics of the participants were summarized using descriptive statistics, the Aspin–Welch test, and Pearson’s chi-square test, as appropriate. Continuous parameters are presented as the means and standard deviations, and categorical variables are expressed as numbers and percentages. The baseline and clinical characteristics of the participants were compared between the two groups of changes in the % slope eGFR. We calculated the effect sizes, including the standardized difference for continuous and categorical variables. The incidence of a ≥10% relative decrease in eGFR per 1000 PY incidence rate difference (IRD), and IRR was calculated using PY of observation and number of events for all participants, and a generalized linear mixed model with binomial distribution and logit link was used to estimate the adjusted OR of change in eGFR. Since specific health checkups are conducted at the same time each year, almost all the participants had contributed at least one PY at follow-up time. This is because the data in this study showed a pattern without variation between the observation periods; almost all events occurred after approximately 365 days from undergoing the health checkup in FY2018. Therefore, we used logistic regression analysis instead of a Cox regression model. The confounding factors included in the multivariate model were preset based on known knowledge. The exposures and confounders included age, sex, HbA1c, BMI, SBP, urine protein, LDL-C, Hb, smoking status, and drinking status. Since non-HDL-C, total bilirubin, alkaline phosphatase, serum creatinine, serum uric acid, and total serum protein were nonessential factors and had many missing values, their data are presented as background characteristics but excluded from the multivariable-adjusted models. All outliers were included in the analysis, and missing values were not processed. In the Japanese health insurance system, in which employers perform the role of insurer, insurance companies often include occupations within the same industry. Thus, there was a possibility of bias in the occupations and regions of the enrollees. Therefore, information on the insurer was added to the mixed effects analysis. Subgroup analyses were performed for the four groups, as previous studies have performed different analyses for diabetes and nondiabetes and for CKD and non-CKD. The adaptive Gauss–Hermite quadrature was used to compute the log-likelihood function. The evaluation criteria for variable selection and the Akaike information criterion were used. Statistical significance was assessed using the 95% CI, and *p* < 0.05 was considered significant. All *P* values were two-tailed. All data were statistically analyzed using Stata 17 (Stata Corp., College Station, Texas, USA).

## Results

### Participant/Descriptive data

**Fig. 2:**
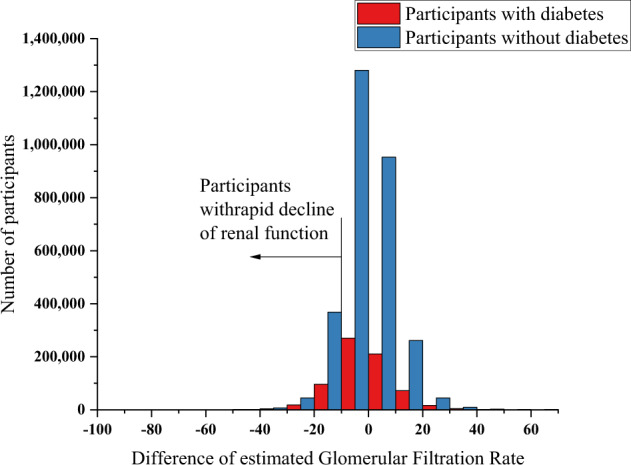
Distribution of the relative decrease in renal function

Of the 3,673,829 participants included in the analysis, 696,952 (18.97%) met the criteria for DM (Fig. 1). Table 1 compares the baseline characteristics of participants with and without diabetes. Among those with diabetes, there were 187,469 (26.9%) women and 509,483 (73.1%) men; among those without diabetes, there were 1,347,187 (45.3%) women and 1629,690 (54.7%) men. More than 88% of those without diabetes tested negative for urinary protein. In contrast, 538,602 (77.5%) patients with diabetes were negative, 81,710 (11.8%) were ±, 46,603 (6.7%) were 1+, 20,720 (3.0%) were 2+, and 7610 (1.1%) were 3+ for urinary protein. Among those with diabetes, the average slope of the eGFR was −0.91 (SD 8.69), the average % slope of the eGFR was −0.65 (SD 17.19), and 120,179 (17.24%) participants had a decrease in eGFR of 10% or more in 1 year, i.e., incidence of 172.22 cases (95% CI 171.24, 173.19) per 1000 PY in a total follow-up time of 697,840 PY Among those without diabetes, the average slope of the eGFR was −0.92 (SD 7.28), the average % slope of the eGFR was −0.75 (SD 15.10), and 422,537 (14.19%) participants had a decrease in eGFR of 10% or more in 1 year, i.e., incidence of 141.50 cases (95% CI 141.07, 141.92) per 1000 PY in a total follow-up time of 2,986,229 PY. The IRD was 30.72 (95% CI 29.66, 31.78) per 1000 PY, and the IRR was 1.22 (95% CI 1.21, 1.22) per 1000 PY (Fig. 2).

**Table 1:** Comparison of characteristics of participants with and without diabetes in the analyzed population

	Participants with diabetes *n* = 696,952		Participants without diabetes *n* = 2,976,877		Std.	*p*-value
	Missing values	Summary	Missing values	Summary	Diff.	
eGFR, mL/min/1.73 m^2^	0	75.16 (17.16)	0	73.82 (13.50)	0.09	<0.001
Age, year	0	60.02 (9.03)	0	57.34 (9.64)	0.28	<0.001
Sex						
Female	0	187,469 (26.9%)	0	1,347,187 (45.3%)	0.39	<0.001
Male		509,483 (73.1%)		1,629,690 (54.7%)	0.39	
Body mass index	20	25.94 (4.46)	35	23.82 (3.81)	0.54	<0.001
Waist circumference, cm	405	90.40 (10.91)	1950	84.37 (10.05)	0.59	<0.001
Systolic BP, mmHg	103,002	130.61 (16.56)	419,258	124.99 (16.79)	0.34	<0.001
Diastolic BP, mmHg	99,163	78.24 (11.18)	403,868	76.34 (11.34)	0.17	<0.001
Triglyceride, mg/dL*	340	152.84 (120.32)	503	121.99 (87.96)	0.32	<0.001
HDL-C, mg/dL^a^	62	55.87 (15.07)	263	62.48 (16.80)	0.40	<0.001
LDL-C, mg/dL^a^	10,279	120.24 (32.17)	39,094	129.36 (31.01)	0.29	<0.001
Aspartate aminotransferase, IU/L	13	27.71 (16.98)	26	23.86 (10.59)	0.32	<0.001
Alanine transaminase, IU/L	11	32.65 (25.20)	49	24.94 (18.07)	0.39	<0.001
Gamma-glutamyl transferase, IU/L	456	54.30 (63.44)	304	39.27 (42.98)	0.32	<0.001
Fasting plasma glucose, mg/dL^b^	159,226	138.43 (36.45)	660,116	98.21 (9.81)	2.22	<0.001
Casual plasma glucose, mg/dL^b^	632,182	144.76 (56.93)	2,722,730	99.91 (17.09)	1.50	<0.001
Hemoglobin A1c, %	0	7.11 (1.17)	0	5.81 (0.20)	2.41	<0.001
Urine glucose						
-	2007	469,311 (67.5%)	6250	2,936,276 (98.8%)	0.92	<0.001
±		24,731 (3.6%)		12,199 (0.4%)	0.23	
1+		35,321 (5.1%)		11,503 (0.4%)	0.29	
2+		40,369 (5.8%)		5918 (0.2%)	0.33	
3+		125,213 (18.0%)		4731 (0.2%)	0.65	
Proteinuria						
-	1707	538,602 (77.5%)	6436	2,614,373 (88.0%)	0.28	<0.001
±		81,710 (11.8%)		260,609 (8.8%)	0.10	
1+		46,603 (6.7%)		73,452 (2.5%)	0.20	
2+		20,720 (3.0%)		17,713 (0.6%)	0.18	
3+		7610 (1.1%)		4294 (0.1%)	0.13	
Hemoglobin, g/dL	196,320	14.93 (1.48)	781,063	14.27 (1.53)	0.43	
Red blood cell count, 10^6^/μL	196,121	488.02 (51.97)	780,473	472.10 (47.66)	0.33	
Anti-hypertensive drugs						
Presence	49	330,184 (47.4%)	395	688,653 (23.1%)	0.53	<0.001
Absence		366,719 (52.6%)		2,287,829 (76.9%)	0.53	
Oral anti-diabetes drugs						
Presence	41	410,537 (58.9%)				<0.001
Absence		286,374 (41.1%)				
Lipid-lowering drugs						
Presence	65	254,775 (36.6%)	420	523,547 (17.6%)	0.44	<0.001
Absence		442,112 (63.4%)		2,452,910 (82.4%)	0.44	
History of stroke						
Yes	35,670	24,035 (3.6%)	155,598	52,534 (1.9%)	0.10	<0.001
No		637,247 (96.4%)		2,768,745 (98.1%)	0.10	
History of heart disease						
Yes	35,497	50,229 (7.6%)	155,643	103,148 (3.7%)	0.17	<0.001
No		611,226 (92.4%)		2,718,086 (96.3%)	0.17	
History of kidney disease						
Yes	38,940	6252 (1.0%)	168,680	13,886 (0.5%)	0.06	<0.001
No		651,760 (99.0%)		2,794,311 (99.5%)	0.06	
History of anemia						
Yes	37,332	36,857 (5.6%)	163,121	294,474 (10.5%)	0.18	<0.001
No		622,763 (94.4%)		2,519,282 (89.5%)	0.18	
Current smoking						
Yes	57	190,016 (27.3%)	446	648,796 (21.8%)	0.13	<0.001
No		506,879 (72.7%)		2,327,635 (78.2%)	0.13	
Frequency of drinking alcohol						
Every day	35,299	178,424 (27.0%)	147,673	660,314 (23.3%)	0.09	<0.001
Not everyday		183,531 (27.7%)		835,240 (29.5%)	0.04	
No alcohol consumption		299,698 (45.3%)		1,333,650 (47.1%)	0.04	

Data are presented as *n* (%) or mean (SD). *Std*. *diff.* denotes standardized difference, *eGFR* estimated glomerular filtration rate, *LDL-C* low-density lipoprotein cholesterol, and *HDL-C* high-density lipoprotein cholesterol

*To convert the values for triglycerides to mmol/L multiplied by 0.01129

^a^To convert values of cholesterol to mmol/L, multiplied by 0.02586

^b^To convert glucose values to mmol/L multiplied by 0.05551

Among those with diabetes and an eGFR of < 60 ml/min/1.73 m^2^ at baseline, the incidence of eGFR was <30 mL/min/1.73 m^2^ with 49.93 cases (95% CI 48.64, 51.25) per 1000 PY during a total follow-up time of 112,474 PY, compared to 11.42 cases (95% CI 11.09, 11.75) per 1000 PY during a total follow-up time of 397,728 PY among those without diabetes. The IRD was 38.51 (95% CI 37.17, 39.86) per 1000 PY, and the IRR was 4.37 (95% CI 4.20, 4.55) per 1000 PY. Among those with diabetes with an eGFR ≥60 mL/min/1.73 m^2^ at baseline, the incidence of an eGFR < 30 mL/min/1.73 m^2^ was 14.69 cases (95% CI 11.89, 18.15) per 100,000 PY during a total follow-up time of 585,367 PY, compared with 6.76 cases (95% CI 5.83, 7.84) per 100,000 PY during a total follow-up time of 2,588,500 PY among those without diabetes. The IRD and IRR were 7.93 (95% CI 4.97, 11.19) and 2.17 (95% CI 1.66, 2.83) per 100,000 PY, respectively.

### Relation of rapid decline in renal function to management goals

Tables 2 and 3 compare the risks of renal function decline between patients with and without diabetes. Participants with an SBP ≥ 120 mmHg and < 130 mmHg had a higher risk of rapid decline than those with an SBP < 120 (OR 1.10, 95% CI 1.07–1.13; *p* < 0.001). The ORs also increased with poorer BP control; the ORs for an SBP > 130 and < 140 mmHg,  > 140 and < 150 mmHg,  > 150 and < 160 mmHg, and ≥160 mmHg were 1.21 (1.17, 1.25; *p* < 0.001), 1.28 (1.21, 1.36; *p* < 0.001), 1.38 (1.33, 1.43; *p* < 0.001), and 1.54 (1.48, 1.61; *p* < 0.001), respectively. The multivariate-adjusted OR for an HbA1c level between 6.2% and 6.9% was slightly lower than that for an HbA1c level < 6.2% (OR 0.96, 95% CI, 0.93, 0.98; *p* = 0.002), and no significant association was found for the group with an HbA1c level between 6.9% and 7.4%. The OR for an HbA1c level between 7.4% and 8.4% was 1.10 (95% CI 1.07, 1.15; *p* < 0.001), and for that between 7.4% and 8.4% was 1.80 (95% CI 1.74, 1.87; *p* < 0.001). The ORs for the rapid decline in renal function increased with increasing levels of urinary protein excretion relative to those with no urinary protein excretion. The adjusted OR of those with plus-minus for urinary protein was 0.99 (0.95, 1.04; *p* = 0.8), with 1 + was 1.15 (1.11, 1.19; *p* < 0.001), 2 +  1.87 (1.78, 1.96; *p* < 0.001), and 3 +  3.37 (3.07, 3.70; *p* < 0.001). Participants with LDL-C levels ≥100 and < 130 mg/dL had a lower risk of rapid decline than those with LDL-C levels < 100 mg/dL (OR 0.90, 95% CI 0.88, 0.92; *p* < 0.001). The ORs also decreased with higher LDL-C levels; the OR for an LDL-C level ≥120 mg/dL was 0.82 (0.80, 0.85; *p* < 0.001). Participants with Hb levels ≥11 and < 13 mg/dL had a higher risk of rapid decline than those with Hb levels ≥13 mg/dL (OR 1.41, 95% CI: 1.33, 1.50; *p* < 0.001). The ORs also increased with lower Hb levels; the OR for Hb levels ≥9 and < 11 mg/dL was 1.73 (95% CI 1.57, 1.92; *p* < 0.001), and that for Hb levels < 9 mg/dL was 1.54 (95% CI 1.18, 2.00; *p* < 0.001). Participants aged ≥60 years had a slightly lower risk of rapid decline in renal function than those aged < 60 years in the univariate analysis, but this was not significant in the multivariate analysis, except for those aged ≥60 and <65 years. Sex and BMI were not significantly associated in the multivariate analysis. In the analysis of those without diabetes, hypertension, severe urinary protein, and anemia were identified as the main risk factors, similar to those with diabetes. Conversely, the results differed in that the ORs for women and obese individuals were higher, while those for elderly and underweight individuals were slightly lower. The results were similar for renal function at baseline, with and without antihypertensive medication use, subgroups of hyperlipidemia medication use, and absolute change in renal function adjusted for baseline renal function (Supplementary Tables 1–5). Regarding the frequency of rapid decline in renal function and urinary protein excretion relative to the class at baseline, the frequency increased with increasing urinary protein excretion among patients with and without diabetes. In contrast, 2–3% of participants with mild and moderate declines in eGFR of 45–60 mL/min/1.73 m^2^ had a rapid decline in renal function, even with negative and plus/minus urinary protein excretion (Table 4).

**Table 2:** Comparison of characteristics of participants with rapid and non-rapid decline

	Participants with diabetes				Participants without diabetes			
	Rapid decline	Non-rapid decline	Std.	*p*-value	Rapid decline	Non-rapid decline	Std.	*p*-value
	*n* = 120,179	*n* = 576,773	diff.*		*n* = 422,537	*n* = 2,554,340	diff.*	
eGFR, mL/min/1.73 m^2^	79.58 (20.92)	74.24 (16.12)	0.31	<0.001	79.43 (15.50)	72.89 (12.91)	0.49	<0.001
Age, year	59.84 (9.15)	60.06 (9.01)	0.02	<0.001	57.18 (9.74)	57.36 (9.62)	0.02	<0.001
Sex								
Female	88,118 (73.3%)	421,365 (73.1%)	0.01	0.06	226,255 (53.5%)	1,403,435 (54.9%)	0.03	<0.001
Male	32,061 (26.7%)	155,408 (26.9%)			196,282 (46.5%)	1,150,905 (45.1%)		
Body mass index	26.08 (4.54)	25.92 (4.44)	0.04	<0.001	23.92 (3.88)	23.81 (3.80)	0.03	<0.001
Waist circumference, cm	90.75 (11.06)	90.32 (10.87)	0.04	<0.001	84.56 (10.17)	84.33 (10.03)	0.02	<0.001
Systolic blood pressure, mmHg	132.61 (17.24)	130.20 (16.39)	0.15	<0.001	126.08 (17.26)	124.81 (16.71)	0.08	<0.001
Diastolic blood pressure, mmHg	78.85 (11.51)	78.11 (11.10)	0.07	<0.001	76.67 (11.58)	76.29 (11.30)	0.03	<0.001
Triglyceride, mg/dL^*^	166.50 (145.08)	149.99 (114.29)	0.14	<0.001	126.78 (100.13)	121.20 (85.76)	0.06	<0.001
HDL-C, mg/dL^a^	55.04 (15.13)	56.05 (15.05)	0.07	<0.001	61.95 (16.77)	62.56 (16.81)	0.04	<0.001
LDL-C, mg/dL^a^	118.51 (32.83)	120.60 (32.02)	0.06	<0.001	126.97 (31.02)	129.76 (30.99)	0.09	<0.001
Aspartate aminotransferase, IU/L	27.73 (17.97)	27.70 (16.76)	0.00	0.5	23.97 (11.29)	23.84 (10.47)	0.01	<0.001
Alanine transaminase, IU/L	32.24 (25.66)	32.74 (25.11)	0.02	<0.001	24.95 (18.34)	24.94 (18.03)	0.00	<0.001
Gamma-glutamyl transferase, IU/L	56.63 (68.80)	53.82 (62.26)	0.04	<0.001	39.97 (44.96)	39.15 (42.65)	0.02	<0.001
Fasting plasma glucose, mg/dL^b^	145.14 (44.91)	137.06 (34.31)	0.22	<0.001	98.08 (9.88)	98.24 (9.80)	0.02	<0.001
Casual plasma glucose, mg/dL^b^	152.97 (66.33)	142.88 (54.38)	0.18	<0.001	100.34 (17.45)	99.83 (17.02)	0.03	<0.001
Hemoglobin A1c, %	7.35 (1.49)	7.05 (1.08)	0.26	<0.001	5.81 (0.20)	5.81 (0.20)	0.00	<0.001
Urine glucose								
-	73,730 (61.6%)	395,581 (68.8%)	0.15	<0.001	415,551 (98.6%)	2,520,725 (98.9%)	0.03	<0.001
±	4971 (4.2%)	19,760 (3.4%)	0.04		2122 (0.5%)	10,077 (0.4%)	0.01	
1+	7539 (6.3%)	27,782 (4.8%)	0.07		2046 (0.5%)	9457 (0.4%)	0.01	
2+	8651 (7.2%)	31,718 (5.5%)	0.07		1107 (0.3%)	4811 (0.2%)	0.02	
3+	24,838 (20.7%)	100,375 (17.4%)	0.08		798 (0.2%)	3933 (0.2%)	0.00	
Proteinuria								
-	86,902 (72.5%)	451,700 (78.5%)	0.14	<0.001	370,918 (88.0%)	2,243,455 (88.0%)	0.00	<0.001
±	13,905 (11.6%)	67,805 (11.8%)	0.01		34,871 (8.3%)	225,738 (8.9%)	0.02	
1+	9384 (7.8%)	37,219 (6.5%)	0.05		10,773 (2.6%)	62,679 (2.5%)	0.01	
2+	6145 (5.1%)	14,575 (2.5%)	0.14		3756 (0.9%)	13,957 (0.5%)	0.05	
3+	3455 (2.9%)	4155 (0.7%)	0.17		1280 (0.3%)	3014 (0.1%)	0.04	
Hemoglobin, g/dL	14.78 (1.55)	14.96 (1.47)	0.12	<0.001	14.13 (1.57)	14.30 (1.52)	0.11	<0.001
Red blood cell count, 10^6^/μL	483.70 (53.97)	488.93 (51.50)	0.10	<0.001	468.22 (48.15)	472.74 (47.55)	0.09	<0.001
Anti-hypertensive drugs								
Presence	60,955 (50.7%)	269,229 (46.7%)	0.08	<0.001	106,835 (25.3%)	581,818 (22.8%)	0.06	<0.001
Absence	59,214 (49.3%)	307,505 (53.3%)			315,652 (74.7%)	1,972,177 (77.2%)		
Oral anti-diabetic drugs								
Presence	72,343 (60.2%)	338,194 (58.6%)	0.03	<0.001	–	–	–	–
Absence	47,828 (39.8%)	238,546 (41.4%)			–	–		
Lipid-lowering drugs								
Presence	43,523 (36.2%)	211,252 (36.6%)	0.01	0.007	74,458 (17.6%)	449,089 (17.6%)	0.00	<0.001
Absence	76,644 (63.8%)	365,468 (63.4%)			348,023 (82.4%)	2,104,887 (82.4%)		
History of stroke								
Yes	4748 (4.2%)	19,287 (3.5%)	0.04	<0.001	8289 (2.1%)	44,245 (1.8%)	0.02	<0.001
No	109,145 (95.8%)	528,102 (96.5%)			391,513 (97.9%)	2,377,232 (98.2%)		
History of heart disease								
Yes	9246 (8.1%)	40,983 (7.5%)	0.02	<0.001	15,669 (3.9%)	87,479 (3.6%)	0.02	<0.001
No	104,690 (91.9%)	506,536 (92.5%)			384,128 (96.1%)	2,333,958 (96.4%)		
Anemia								
Yes	6898 (6.1%)	29,959 (5.5%)	0.03	<0.001	44,176 (11.1%)	250,298 (10.4%)	0.02	<0.001
No	106,611 (93.9%)	516,152 (94.5%)			354,167 (88.9%)	2,165,115 (89.6%)		

Data are presented as *n* (%) or mean (SD). *Std*. *diff*. denotes standardized difference, *eGFR* estimated glomerular filtration rate, *LDL-C*low-density lipoprotein cholesterol, *HDL-C* high-density lipoprotein cholesterol, *Rapid decline* %slope in estimated glomerular filtration rate is over −10%, and *non-rapid decline* %slope in estimated glomerular filtration rate is less than −10%

^*^To convert the values for triglycerides to mmol/L multiplied by 0.01129

^a^To convert values of cholesterol to mmol/L, multiplied by 0.02586

^b^To convert glucose values to mmol/L multiplied by 0.05551

**Table 3:** Odds ratio of rapid decline in renal function by management goals

	Participants with diabetes *n* = 297,972				Participants without diabetes *n* = 1,311,993			
	Crude		Adjusted		Crude		Adjusted	
	OR (95% CI)	*p*-value	OR (95% CI)	*p*-value	OR (95%CI)	*p*-value	OR (95% CI)	*p*-value
Age, year								
Less than 60	Reference		Reference		Reference		Reference	
≥60 to < 65	0.92 (0.91, 0.94)	<0.001	0.96 (0.93,0.99)	0.0	0.94 (0.93, 0.95)	<0.001	0.93 (0.91, 0.95)	<0.001
≥65 to < 70	0.93 (0.92, 0.95)	<0.001	0.98 (0.96,1.00)	0.1	0.97 (0.95, 0.99)	0.003	0.94 (0.91, 0.97)	<0.001
≥70	0.96 (0.94, 0.98)	<0.001	1.01 (0.97,1.05)	0.6	1.02 (1.00, 1.03)	0.07	0.97 (0.94, 1.00)	0.05
Gender								
Man	Reference		Reference		Reference		Reference	
Woman	0.99 (0.97, 1.00)	0.07	1.00 (0.97,1.03)	0.9	1.07 (1.05, 1.09)	<0.001	1.05 (1.04, 1.07)	<0.001
Hemoglobin A1c, %								
Less than 6.2	Reference		Reference		Reference		Reference	
≥6.2 to < 6.9	0.93 (0.91, 0.94)	<0.001	0.96 (0.93, 0.98)	0.002	1.03 (1.02, 1.04)	<0.001	1.01 (0.99, 1.03)	0.3
≥6.9 to < 7.4	0.97 (0.95, 0.99)	0.003	0.99 (0.96, 1.02)	0.5	–	–	–	–
≥7.4 to < 8.4	1.12 (1.09, 1.15)	<0.001	1.11 (1.07, 1.15)	<0.001	–	–	–	–
≥8.4	1.83 (1.79, 1.87)	<0.001	1.80 (1.74, 1.87)	<0.001	–	–	–	–
Body mass index, kg/m^2^								
Less than 18.5	1.00 (0.95, 1.06)	0.9	0.96 (0.89, 1.03)	0.2	1.00 (0.99, 1.02)	0.6	0.96 (0.94, 0.98)	<0.001
≥18.5 to < 25	Reference		Reference		Reference		Reference	
≥25	1.07 (1.05, 1.08)	<0.001	1.00 (0.98, 1.02)	0.9	1.05 (1.04, 1.07)	<0.001	1.04 (1.02, 1.05)	<0.001
Systolic blood pressure, mmHg								
Less than 120	Reference		Reference		Reference		Reference	
≥120 to < 130	1.10 (1.08, 1.12)	<0.001	1.10 (1.07, 1.13)	<0.001	1.05 (1.04, 1.07)	<0.001	1.07 (1.06, 1.08)	<0.001
≥130 to < 140	1.21 (1.19, 1.24)	<0.001	1.21 (1.17, 1.25)	<0.001	1.11 (1.09, 1.12)	<0.001	1.13 (1.11, 1.15)	<0.001
≥140 to < 150	1.33 (1.27, 1.38)	<0.001	1.28 (1.21, 1.36)	<0.001	1.18 (1.16, 1.20)	<0.001	1.21 (1.18, 1.23)	<0.001
≥150 to < 160	1.44 (1.41, 1.48)	<0.001	1.38 (1.33, 1.43)	<0.001	1.25 (1.22, 1.29)	<0.001	1.26 (1.22, 1.30)	<0.001
≥160	1.68 (1.63, 1.73)	<0.001	1.54 (1.48, 1.61)	<0.001	1.37 (1.34, 1.41)	<0.001	1.37 (1.34, 1.41)	<0.001
Proteinuria								
-	Reference		Reference		Reference		Reference	
±	1.05 (1.02, 1.09)	0.002	0.99 (0.95,1.04)	0.8	0.91 (0.89, 0.93)	<0.001	0.89 (0.86, 0.91)	<0.001
1 +	1.30 (1.27, 1.33)	<0.001	1.15 (1.11,1.19)	<0.001	1.02 (0.99, 1.04)	0.2	0.96 (0.92, 1.01)	0.1
2 +	2.18 (2.07, 2.29)	<0.001	1.87 (1.78,1.96)	<0.001	1.61 (1.56, 1.66)	<0.001	1.53 (1.46, 1.61)	<0.001
3 +	4.30 (3.91, 4.73)	<0.001	3.37 (3.07,3.70)	<0.001	2.54 (2.29, 2.82)	<0.001	2.38 (2.10, 2.71)	<0.001
LDL-C, mg/dL								
Less than 100	Reference		Reference		Reference		Reference	
≥100 to < 120	0.90 (0.88, 0.91)	<0.001	0.90 (0.88, 0.92)	<0.001	0.90 (0.89, 0.91)	<0.001	0.90 (0.89, 0.91)	<0.001
≥120	0.85 (0.84, 0.86)	<0.001	0.82 (0.80, 0.85)	<0.001	0.81 (0.80, 0.82)	<0.001	0.81 (0.80, 0.82)	<0.001
Hemoglobin, g/dL								
Less than 9	1.55 (1.35, 1.78)	<0.001	1.54 (1.18, 2.00)	<0.001	1.66 (1.56, 1.76)	<0.001	1.63 (1.51, 1.77)	<0.001
≥9 to < 11	1.83 (1.70, 1.97)	<0.001	1.73 (1.57, 1.92)	<0.001	1.32 (1.29, 1.35)	<0.001	1.27 (1.23, 1.31)	<0.001
≥11 to < 13	1.37 (1.30, 1.45)	<0.001	1.41 (1.33, 1.50)	<0.001	1.22 (1.19, 1.25)	<0.001	1.24 (1.21, 1.27)	<0.001
≥13	Reference		Reference		Reference		Reference	
Variance in insurer level			0.032 (0.023, 0.044)				0.079 (0.064, 0.096)	
Number of insurers			1754				2176	

*Rapid decline* denotes %slope in estimated glomerular filtration rate is over −10%, *non-rapid decline* %slope in estimated glomerular filtration rate is less than −10%, *OR* odds ratio, *adjusted* adjusted for current smoking and quantity of drinking alcohol, *95%CI* 95% confidence intervals, and *LDL-C* low-density lipoprotein cholesterol

**Table 4:** Prevalence of renal function classification and urinary protein excretion classification

		Proteinuria						
CKD	eGFR	-	±	1 +	2 +	3 +	Missing	Total
Grade1^*^	90 or more	93,150 (13.4%)	14,399 (2.07%)	8091 (1.16%)	2799 (0.4%)	614 (0.09%)	369 (0.05%)	119,422 (17.12%)
Proportion		25,839 (27.74%)	4146 (28.79%)	2430 (30.03%)	1041 (37.19%)	248 (40.39%)	109 (29.54%)	33,813 (28.31%)
Grade2	≥60 to <90	370,461 (53.28%)	53,850 (7.75%)	27,637 (3.98%)	9868 (1.42%)	2706 (0.39%)	761 (0.11%)	465,283 (66.76%)
Proportion		30,141 (8.14%)	4622 (8.58%)	3074 (11.12%)	1651 (16.73%)	621 (22.95%)	80 (10.51%)	40,189 (8.64%)
Grade3a	≥45 to <60	67,019 (9.64%)	11,271 (1.62%)	7963 (1.15%)	4528 (0.65%)	1691 (0.24%)	161 (0.02%)	92,633 (13.29%)
Proportion		1522 (2.27%)	345 (3.06)	415 (5.21%)	458 (10.11%)	319 (18.86%)	12 (7.45%)	3071 (3.32%)
Grade3b	≥30 to < 45	7330 (1.05%)	1921 (0.28%)	2251 (0.32%)	2217 (0.32%)	1306 (0.19%)	43 (0.01%)	15,068 (2.16%)
Proportion		130 (1.77%)	33 (1.72%)	65 (2.89%)	176 (7.94%)	275 (21.06%)	3 (6.98%)	682 (4.53%)
Grade4	≥15 to <30	529 (0.08%)	220 (0.03%)	474 (0.07%)	852 (0.12%)	819 (0.12%)	12 (0.00%)	2906 (0.42%)
Proportion		6 (1.13%)	2 (0.91%)	11 (2.32%)	56 (6.92%)	159 (19.41%)	1 (8.33%)	238 (8.19%)
Grade5	<15	113 (0.02%)	49 (0.01%)	187 (0.03%)	456 (0.07%)	474 (0.07%)	361 (0.05%)	1640 (0.24%)
Proportion		0 (0.00%)	0 (0.00%)	0 (0.00%)	1 (0.22%)	3 (0.63%)	0 (0.00%)	4 (0.24%)
Total		538,602 (77.47%)	81,710 (11.75%)	46,603 (6.7%)	20,720 (2.98%)	7610 (1.09%)	1707 (0.24%)	696,952 (100%)
Proportion		57,638 (10.7%)	9148 (11.2%)	5995 (12.86%)	3386 (16.34%)	1625 (21.35%)	205 (12.01%)	77,997 (11.19%)
Grade1^a^	90 or more	295,325 (9.94%)	28,279 (0.95%)	7056 (0.24%)	1266 (0.04%)	236 (0.01%)	1167 (0.04%)	333,329 (11.20%)
Proportion		81,158 (27.48%)	7531 (26.63%)	1870 (26.5%)	367 (28.99%)	50 (21.19%)	283 (24.25%)	91,259 (27.38%)
Grade2	≥60 to <90	1,987,950 (66.92%)	194,302 (6.54%)	49,234 (1.66%)	9417 (0.32%)	1801 (0.06%)	4519 (0.15%)	2,247,223 (75.49%)
Proportion		135,224 (6.8%)	12,683 (6.53%)	3492 (7.09%)	895 (9.5%)	232 (12.88%)	307 (6.79%)	152,833 (6.80%)
Grade3a	≥45 to < 60	312,961 (10.54%)	34,673 (1.17%)	13,827 (0.47%)	4328 (0.15%)	1089 (0.04%)	442 (0.00%)	367,320 (12.34%)
Proportion		3841 (1.23%)	564 (1.63%)	358 (2.59%)	227 (5.24%)	106 (9.73%)	4 (0.9%)	5100 (1.39%)
Grade3b	≥30 to < 45	16,826 (0.57%)	2945 (0.1%)	2647 (0.09%)	1776 (0.06%)	688 (0.02%)	38 (0.00%)	24,920 (0.84%)
Proportion		174 (1.03%)	47 (1.6%)	62 (2.34%)	91 (5.12%)	66 (9.59%)	1 (2.63%)	441 (1.77%)
Grade4	≥15 to < 30	884 (0.03%)	316 (0.01%)	536 (0.02%)	665 (0.02%)	339 (0.01%)	9 (0.00%)	2749 (0.09%)
Proportion		8 (0.9%)	2 (0.63%)	6 (1.12%)	29 (4.36%)	25 (7.37%)	2 (22.22%)	72 (2.62%)
Grade5	<15	427 (0.01%)	94 (0.00%)	152 (0.01%)	261 (0.01%)	141 (0.00%)	261 (0.01%)	1336 (0.04%)
Proportion		0 (0.00%)	0 (0.00%)	0 (0.00%)	1 (0.38%)	0 (0.00%)	0 (0.00%)	1 (0.07%)
Total		2,614,373 (88.01%)	260,609 (8.77%)	73,452 (2.47%)	17,713 (0.6%)	4294 (0.14%)	6436 (0.22%)	2,976,877 (100%)
Proportion		220,405 (8.43%)	20,827 (7.99%)	5788 (7.88%)	1610 (9.09%)	479 (11.16%)	597 (9.28%)	249,706 (8.39%)

*CKD* denotes chronic kidney disease grading, *Proportion* the proportion of %slope in estimated glomerular filtration rate is over −10%, and *eGFR* estimated glomerular filtration rate (mL/min/1.73m^2^)

^*^Participants with diabetes in fiscal year 2018

^a^Participants without diabetes in fiscal year 2018

## Discussion

This study examined the rapid decline in renal function based on the largest complete set of information, including information from nonmedical institutions in Japan’s national database of medical insurance records. We showed that 20.83% of patients with DM had a rapid decline in renal function within the observation period, approximately 1.2 times that of patients without diabetes. Compared with previous studies, the slope of eGFR was similar for both patients with and without diabetes [11, 12]. Participants included in the higher SBP group compared with those included in the lower group had a 37–54% upper relative risk of the primary outcome regardless of diabetic status; moreover, the high level of urinary protein excretion group had higher rates of the primary outcome than the lower level of urinary protein excretion group, the lower LDL-C group had a 1.5–2% lower relative risk of the primary outcome than the higher LDL-C group, and the lower Hb group had a 2.4–7.3% higher relative risk of the primary outcome than the higher Hb group. These benefits with respect to the primary outcome were consistent across all prespecified subgroups, including participants with an eGFR > 60 mL/min/1.73 m^2^. Among participants without CKD at baseline, a decreased eGFR of ≥30% to a value of <60 mL/min/1.73 m^2^ occurred more frequently in the diabetic group than in the nondiabetic group.

These results add substantially to the evidence on the benefits of lowering SBP, regardless of whether the patients have diabetes or CKD. Trials such as the SPRINT showed the benefits of lowering SBP to <120 mmHg [21, 22]. However, these studies evaluated renal outcomes and did not observe a rapid decline. Additionally, the scope of coverage was limited. Poor BP control reportedly contributes to a rapid decline in renal function [14, 23–25]. This study’s results are similar to those of previous studies. Blindly lowering the blood pressure of elderly individuals should also consider the emergence of other complications. Lowering blood pressure too much may induce rapid renal dysfunction due to the rapid decline in intraglomerular pressure, especially in elderly individuals. Therefore, a subgroup analysis was conducted on the effect of blood pressure on the rapid decline in renal function by age. In both strata, there was no significant difference in the direction of risk for blood pressure lower than 120 mmHg versus ≥120 mmHg or ≤130 mmHg (Supplementary Fig. 1). The SPRINT showed that stricter blood pressure control resulted in an increased rate of acute renal failure, but our data did not show such a trend. The fact that our participants were between 40 and 74 years of age may account for this difference. Furthermore, this study analyzed real-world data and was characterized by the evaluation of blood pressure in the absence of interventions for blood pressure control. Among older participants not included in this study, the possibility that tight blood pressure control could increase the risk of other complications is important and should be interpreted with caution; however, this could not be further explored in this study. This study additionally found that the risk increased with the degree of BP control and provided evidence of benefits for an even lower SBP target than that currently recommended in guidelines [4, 19, 20, 22]. The differences in the rapid decline in renal function may be related to a reversible intrarenal hemodynamic effect of the greater reduction in BP and increased use of diuretics, angiotensin-converting enzyme inhibitors, and angiotensin-receptor blockers [22, 26]; however, the lack of information on the prescription of antihypertensive drugs in this study makes it difficult to clarify these mechanisms.

Consistent with previous studies, poor glycemic control HbA1c level > 7.4% is an independent risk factor for the rapid decline in renal function. Previous studies have shown that the effect on renal function decline differs depending not only on the HbA1c control status but also on what drugs are used to treat diabetes [26]. Prescribing appropriate antidiabetic drugs such as sodium-glucose cotransporter 2 inhibitor therapy may lessen renal function decline.

Furthermore, poor HbA1c control reportedly contributes to a rapid decline in renal function [4, 19, 20, 22]. Moreover, treatment with glucagon-like peptide-1 receptor agonists reportedly reduces albuminuria [27]. The results of this study are similar to those of previous studies [13, 15] but showed a decreased risk of a rapid decline in renal function in the group with HbA1c levels ≥6.2% and ≤7.4%. Repetitive hypoglycemia may exacerbate microvascular damage, including DN, and thus may put patients at risk of reduced renal function. In the present results, no increase in risk was observed at 7.0–7.4 g/dL. In general, management goals for complication prevention are set from a long-term perspective. In this study, events were evaluated over a period of 1 year; hence, no significant results were obtained in groups other than those with severely poor control. However, the lack of information on the prescription of diabetes medications in this study made it difficult to distinguish between the relatively mild and strictly controlled groups, making a further detailed examination of the results difficult. With the currently available data, there is no evidence of a rapid decline in renal function with strict control of HbA1c; however, the possibility of a long-term adverse renal outcome cannot be excluded. These observations need to be further explored in analyses that incorporate more clinical outcomes and longer follow-up periods.

An increase in urinary protein excretion is associated with a rapid decline in renal function [14, 26, 28]. Herein, the results were more prominent among participants with DM than among those without DM. This is consistent with the rapid decline in renal function without urinary protein excretion recently reported in DKD [2, 29]. The prevalence of individuals who fell into the category of high urinary protein excretion was very low: only 4.07% among patients with DM and <1% among patients without DM (Table 4) due to the limited number of participants with health checkup data. Therefore, it was difficult to conduct a detailed analysis without a large dataset. This study included approximately 28,330 participants with diabetes and 22,007 without diabetes with high urinary protein excretion, which is considered valuable.

Anemia is an independent risk factor for renal function decline, and improvement of anemia leads to a reduction in renal function [30]. Although the mean Hb levels among both participants with and without diabetes in this study did not meet the definition of anemia, there were no previous reports of anemia, with Hb < 13 g/dL being a contributing factor to a higher incidence of rapid renal function decline [3]. The relationship between hemoglobin and renal function must be differentiated from renal anemia, which is caused by reduced renal function. Therefore, we compared baseline renal function relative to four categories of hemoglobin values. We found that the highest age-adjusted baseline renal function was below 9 g/dL and that the impact of anemia on baseline renal function differed from the impact of anemia on the rapid decline over 1 year (Supplementary Table 6). These findings suggest that anemia at baseline may be the cause of the rapid decline in renal function.

### Limitations/generalizability

The strengths of our study include the large sample size, diversity of the population, and its success in achieving an assessment of the rapid decline in renal function for approximately 1 year in a population with or without medical care rather than in a medical institution-based population. Therefore, the results obtained are useful for proposing management strategies to prevent CKD progression in populations with or without DM.

The findings of this study should be interpreted in the context of some potential limitations. First is the lack of generalizability to populations excluded from this study, including participants who receive a public service that guarantees the minimum standard of living for poor people, called welfare; those who have not received the specified medical checkup even though the health checkup is free for all; and those aged < 40 years and >75 years. Moreover, the measurement of eGFR was institutionalized in specific health checkups starting in FY2018 in Japan. Therefore, the follow-up period for renal function assessment based on eGFR was only approximately 1 year. The fact that the specified checkups can be performed only once a year and thus cannot account for fluctuations in measurements is a limitation. Finally, information about dietary intake, such as sodium intake, and treatment information, especially drug prescription details, was unavailable in this study. Research on the rapid decline in renal function in Japan is still in its infancy. We believe that further studies with long-term follow-up and more clinical outcomes are needed.

### Asian perspectives

Asia has a higher prevalence of diabetes than other regions. China ranks first in terms of its diabetic population, and Japan ranks ninth, making Asia the region with the highest number of diabetic individuals worldwide [31]. Furthermore, the aging population of Asia is increasing at an unmatched speed. Because the prevalence of diabetes increases with age [32], the prevalence of diabetes will increase substantially in Asia, and Asia will house the largest number of diabetic patients worldwide by 2045 [31].

In addition to diabetes, hypertension is more common in most Asian countries than in the West [33, 34]. The high prevalence and poor glycemic and blood pressure control compound the severity of this problem [33]. More countermeasures are needed that consider not only lifestyle habits but also genetic factors such as salt intake, higher salt sensitivity, and possession of specific genes for diabetes [35–37]. Regarding blood pressure control among diabetic patients, research is being conducted on the usefulness of stricter standards than those set by existing guidelines [21, 25]. Appropriate blood pressure control among diabetic patients prevents various complications, such as cardiovascular disease, cerebrovascular disease, and chronic renal failure [24, 25]. Health care policy-makers across Asia are called upon to help take action to improve the lifestyles of those living with diabetes and those at risk of developing diabetes.

### Future perspectives

In recent years, various diabetes treatment guidelines have been proposed and have become available [20, 38–41]. In particular, the latest diabetes treatment guidelines emphasize the usefulness of SGLT2, but it is only an evaluation based on the additional effect of angiotensin-converting enzyme inhibitors and angiotensin II receptor blockers [26]. Asian diabetic patients have lower mortality from complications of cardiovascular disease than Western diabetic patients [9, 42]. In the future, it is desirable to propose and update guidelines that reflect Asian patient characteristics.

In conclusion, the rapid decline in renal function for approximately 1 year was associated with classical risks such as systolic BP > 120 mmHg, poor glycemic control (HbA1c > 7.4%), increased urinary protein excretion, and blood hemoglobin levels < 13 mg/dL regardless of DM or non-DM and CKD or non-CKD, resulting in lower rates of decline in rapid renal function based on large real-world data in the national medical insurance database in Japan. These results will be useful for establishing effective health guidance and guidelines for CKD prevention.

## Supplementary Information

Supplementary Table

Checklist

## Data Availability

The data may be obtained from a third party and are not publicly available. The data used in this study are from the MHLW in Japan; therefore, users of these data are strictly limited to those who obtain official permission from the MHLW in accordance with Japanese Article 33 (Provision of Questionnaire Information) of the Statistics Act, by the Statistics Bureau, Ministry of Internal Affairs and Communications. Qualified researchers who would like to request access to data should contact the Statistics and Information Department of the MHLW.
