# Supplementary material for: Current status of the rapid decline in renal function due to diabetes mellitus and its associated factors: analysis using the National Database of Health Checkups in Japan
Source: Hypertens Res. 2023 Feb 2;46(5):1075–89. doi: 10.1038/s41440-023-01185-2 (PMC10164644; doi:10.1038/s41440-023-01185-2)
Supplement: Supplementary file 1 — Supplementary Table [file 41440_2023_1185_MOESM1_ESM.docx]

**Supplemental Table 1. Comparison of the baseline characteristics of participants who underwent examination in both years and those who were not re-examined in FY 2018.**

|  |  | **Participants with diabetes** | | |  | **Participants without diabetes** | | |
| --- | --- | --- | --- | --- | --- | --- | --- | --- |
|  |  | **Participants** | **Non-participants** | **Std** |  | **Participants** | **Non-participants** | **Std** |
|  |  | **n = 696,952** | **n = 274,347** | **diff** |  | **n = 2,976,877** | **n = 1,122,873** | **diff** |
| **eGFR^*^, mL/min/1.73m^2^** | | 75.16 (17.16) | 73.47 (17.85) | 0.10 |  | 73.82 (13.50) | 72.90 (13.98) | 0.07 |
| **Age** |  | 60.02 (9.03) | 63.27 (8.84) | 0.36 |  | 57.34 (9.64) | 60.47 (9.92) | 0.32 |
| **Sex** | |  |  |  |  |  |  |  |
|  | **Woman** | 187,469 (26.9%) | 89,155 (32.5%) | 0.12 |  | 1,347,187 (45.3%) | 511,973 (45.6%) | 0.18 |
|  | **Man** | 509,483 (73.1%) | 185,192 (67.5%) | 0.12 |  | 1,629,690 (54.7%) | 610,900 (54.4%) | 0.18 |
| **Body mass index** | | 25.94 (4.46) | 25.50 (4.31) | 0.10 |  | 23.82 (3.81) | 23.56 (3.76) | 0.07 |
| **Waist circumference, cm** | | 90.40 (10.91) | 89.62 (10.67) | 0.07 |  | 84.37 (10.05) | 83.96 (10.01) | 0.04 |
| **Systolic BP, mmHg** | | 130.61 (16.56) | 132.13 (17.28) | 0.09 |  | 124.99 (16.79) | 126.22 (17.52) | 0.07 |
| **Diastolic BP, mmHg** | | 78.24 (11.18) | 77.56 (11.41) | 0.06 |  | 76.34 (11.34) | 75.93 (11.44) | 0.04 |
| **Triglyceride, mg/dl^*^** | | 152.84 (120.32) | 150.46 (117.64) | 0.02 |  | 121.99 (87.96) | 119.72 (83.94) | 0.03 |
| **HDL-C, mg/dl^†^** | | 55.87 (15.07) | 56.59 (15.44) | 0.05 |  | 62.48 (16.80) | 63.69 (17.03) | 0.07 |
| **LD-C, mg/dl^†^** | | 120.24 (32.17) | 119.47 (32.88) | 0.02 |  | 129.36 (31.01) | 129.40 (31.65) | 0.00 |
| **Non-HDL-C, mg/dl^†^** | | 146.60 (37.45) | 145.29 (38.18) | 0.03 |  | 150.56 (34.69) | 150.37 (35.00) | 0.01 |
| **Total bilirubin, mg/dl^‡^** | | 0.81 (0.34) | 0.76 (0.32) | 0.15 |  | 0.80 (0.33) | 0.78 (0.31) | 0.06 |
| **Aspartate aminotransferase, IU/L** | | 27.71 (16.98) | 27.43 (17.39) | 0.02 |  | 23.86 (10.59) | 23.81 (10.62) | 0.00 |
| **Alanine transaminase, IU/L** | | 32.65 (25.20) | 30.41 (23.75) | 0.09 |  | 24.94 (18.07) | 23.37 (16.86) | 0.09 |
| **Gamma-glutamyl transferase, IU/L** | | 54.30 (63.44) | 52.52 (65.85) | 0.03 |  | 39.27 (42.98) | 36.76 (41.70) | 0.06 |
| **Alkaline Phosphatase, IU/L** | | 222.91 (69.57) | 224.38 (61.13) | 0.02 |  | 206.04 (56.51) | 211.67 (60.91) | 0.10 |
| **Serum creatinine, mg/dL^§^** | | 0.81 (0.41) | 0.82 (0.47) | 0.02 |  | 0.77 (0.22) | 0.75 (0.24) | 0.09 |
| **Serum uric acid, mg/dL^\|\|^** | | 5.58 (1.32) | 5.62 (1.34) | 0.03 |  | 5.56 (1.38) | 5.67 (1.49) | 0.08 |
| **Total serum protein, g/dL** | | 7.26 (0.40) | 7.26 (0.44) | 0.00 |  | 7.17 (0.40) | 7.19 (0.40) | 0.05 |
| **Serum albumin, g/dL** | | 4.40 (0.28) | 4.36 (0.32) | 0.14 |  | 4.38 (0.27) | 4.36 (0.28) | 0.07 |
| **Fasting plasma glucose level, mg/dL^¶^** | | 138.43 (36.45) | 138.81 (38.07) | 0.01 |  | 98.21 (9.81) | 97.74 (9.88) | 0.05 |
| **Blood glucose anytime, mg/dL^¶^** | | 144.76 (56.93) | 148.69 (61.47) | 0.07 |  | 99.91 (17.09) | 100.03 (17.34) | 0.01 |
| **Hemoglobin A1c, %** | | 7.11 (1.17) | 7.12 (1.22) | 0.01 |  | 5.81 (0.20) | 5.80 (0.20) | 0.05 |
| **Urine glucose** | |  |  |  |  |  |  |  |
|  | **-** | 469,311 (67.5%) | 186,989 (68.4%) | 0.02 |  | 2,936,276 (98.8%) | 1,106,770 (98.8%) | 0.00 |
|  | **±** | 24,731 (3.6%) | 10,087 (3.7%) | 0.01 |  | 12,199 (0.4%) | 4,813 (0.4%) | 0.00 |
|  | **1+** | 35,321 (5.1%) | 14,232 (5.2%) | 0.00 |  | 11,503 (0.4%) | 4,399 (0.4%) | 0.00 |
|  | **2+** | 40,369 (5.8%) | 15,805 (5.8%) | 0.00 |  | 5,918 (0.2%) | 2,355 (0.2%) | 0.00 |
|  | **3+** | 125,213 (18.0%) | 46,190 (16.9%) | 0.03 |  | 4,731 (0.2%) | 1,931 (0.2%) | 0.00 |
| **Urine protein** | |  |  |  |  |  |  |  |
|  | **-** | 538,602 (77.5%) | 206,845 (75.6%) | 0.04 |  | 2,614,373 (88.0%) | 980,233 (87.5%) | 0.02 |
|  | **±** | 81,710 (11.8%) | 33,182 (12.1%) | 0.01 |  | 260,609 (8.8%) | 99,375 (8.9%) | 0.00 |
|  | **1+** | 46,603 (6.7%) | 19,979 (7.3%) | 0.02 |  | 73,452 (2.5%) | 30,298 (2.7%) | 0.01 |
|  | **2+** | 20,720 (3.0%) | 9,429 (3.4%) | 0.02 |  | 17,713 (0.6%) | 8,034 (0.7%) | 0.01 |
|  | **3+** | 7,610 (1.1%) | 4,026 (1.5%) | 0.04 |  | 4,294 (0.1%) | 2,257 (0.2%) | 0.03 |
| **Hemoglobin, g/dL** | | 14.93 (1.48) | 14.69 (1.55) | 0.16 |  | 14.27 (1.53) | 14.04 (1.55) | 0.15 |
| **Red blood cell count, 10^6^/μL** | | 488.02 (51.97) | 479.19 (54.85) | 0.17 |  | 472.10 (47.66) | 464.12 (49.52) | 0.17 |
| **Anti-hypertensive drugs** | |  |  |  |  |  |  |  |
|  | **Presence** | 330,184 (47.4%) | 136,082 (49.6%) | 0.04 |  | 688,653 (23.1%) | 283,052 (25.2%) | 0.05 |
|  | **Absence** | 366,719 (52.6%) | 138,210 (50.4%) | 0.04 |  | 2,287,829 (76.9%) | 839,538 (74.8%) | 0.05 |
| **Oral anti-diabetes drugs** | |  |  |  |  |  |  |  |
|  | **Presence** | 410,537 (58.9%) | 160,720 (58.6%) | 0.01 |  | - | - |  |
|  | **Absence** | 286,374 (41.1%) | 113,580 (41.4%) | 0.01 |  | - | - |  |
| **Lipid-lowering drugs** | |  |  |  |  |  |  |  |
|  | **Presence** | 254,775 (36.6%) | 101,599 (37.0%) | 0.01 |  | 523,547 (17.6%) | 209,789 (18.7%) | 0.03 |
|  | **Absence** | 442,112 (63.4%) | 172,675 (63.0%) | 0.01 |  | 2,452,910 (82.4%) | 912,774 (81.3%) | 0.03 |
| **History of stroke** | |  |  |  |  |  |  |  |
|  | **Presence** | 24,035 (3.6%) | 12,796 (4.9%) | 0.06 |  | 52,534 (1.9%) | 26,255 (2.5%) | 0.04 |
|  | **Absence** | 637,247 (96.4%) | 247,471 (95.1%) | 0.06 |  | 2,768,745 (98.1%) | 1,038,408 (97.5%) | 0.04 |
| **History of heart disease** | |  |  |  |  |  |  |  |
|  | **Presence** | 50,229 (7.6%) | 23,473 (9.0%) | 0.05 |  | 103,148 (3.7%) | 48,467 (4.6%) | 0.05 |
|  | **Absence** | 611,226 (92.4%) | 236,786 (91.0%) | 0.05 |  | 2,718,086 (96.3%) | 1,016,038 (95.4%) | 0.05 |
| **History of kidney disease** | |  |  |  |  |  |  |  |
|  | **Presence** | 6,252 (1.0%) | 3,229 (1.2%) | 0.02 |  | 13,886 (0.5%) | 6,549 (0.6%) | 0.01 |
|  | **Absence** | 651,760 (99.0%) | 256,394 (98.8%) | 0.02 |  | 2,794,311 (99.5%) | 1,056,144 (99.4%) | 0.01 |
| **History of anemia** | |  |  |  |  |  |  |  |
|  | **Presence** | 36,857 (5.6%) | 16,441 (6.3%) | 0.03 |  | 294,474 (10.5%) | 125,975 (11.9%) | 0.04 |
|  | **Absence** | 622,763 (94.4%) | 242,696 (93.7%) | 0.03 |  | 2,519,282 (89.5%) | 934,566 (88.1%) | 0.04 |
| **Current smoking, No. (%)** | |  |  |  |  |  |  |  |
|  | **Presence** | 190,016 (27.3%) | 66,490 (24.2%) | 0.07 |  | 648,796 (21.8%) | 199,341 (17.8%) | 0.10 |
|  | **Absence** | 506,879 (72.7%) | 207,809 (75.8%) | 0.07 |  | 2,327,635 (78.2%) | 923,233 (82.2%) | 0.10 |
| **Frequency of drinking alcohol, No. (%)** | | |  |  |  |  |  |  |
|  | **Every day** | 178,424 (27.0%) | 69,511 (26.7%) | 0.01 |  | 660,314 (23.3%) | 232,328 (21.7%) | 0.04 |
|  | **Not everyday** | 183,531 (27.7%) | 66,601 (25.6%) | 0.05 |  | 835,240 (29.5%) | 292,630 (27.4%) | 0.05 |
|  | **No drinking** | 299,698 (45.3%) | 124,510 (47.8%) | 0.05 |  | 1,333,650 (47.1%) | 544,149 (50.9%) | 0.08 |
| **Quantity of drinking alcohol, No. (%)** | | |  |  |  |  |  |  |
|  | **Less than 21.6 g/day** | 250,001 (50.4%) | 103,630 (54.2%) | 0.08 |  | 1,222,914 (58.5%) | 485,189 (63.3%) | 0.10 |
|  | **21.6 g/day to 43.2 g/day** | 146,765 (29.6%) | 53,733 (28.1%) | 0.03 |  | 563,080 (26.9%) | 187,087 (24.4%) | 0.06 |
|  | **43.2 g/day to 64.8 g/day** | 73,458 (14.8%) | 25,398 (13.3%) | 0.04 |  | 230,571 (11.0%) | 71,904 (9.4%) | 0.05 |
|  | **Over 64.8g/day** | 26,198 (5.3%) | 8,427 (4.4%) | 0.04 |  | 74,378 (3.6%) | 22,171 (2.9%) | 0.04 |

Data are presented as n (%) or mean (SD).

Std. diff, standardized difference.

*eGFR, estimated glomerular filtration rate.

†LDL-C, low-density lipoprotein cholesterol.

HDL, high-density lipoprotein cholesterol.

Non-HDL, non-high-density lipoprotein cholesterol.

*To convert the values for triglycerides to mmol/L multiplied by 0.01129.

†To convert values of cholesterol to mmol/L, multiplied by 0.02586.

‡To convert values of total bilirubin to μmol/L, multiply by 17.10.

§To convert creatinine values to μmol/L, multiply by 88.4.

||To convert uric acid values to μmol/L, multiplied by 59.48.

¶To convert glucose values to mmol/L multiplied by 0.05551.

**Supplemental Table 2. Odds ratio of rapid decline in renal function according to subgroups (baseline-** **estimated glomerular filtration rate).**

|  |  | **Participants with diabetes** | | | |  | **Participants without diabetes** | | | |
| --- | --- | --- | --- | --- | --- | --- | --- | --- | --- | --- |
|  |  | **eGFR^*^ < 60**  **n = 42,895** | | **eGFR ≥ 60**  **n = 255,077** | |  | **eGFR < 60**  **n= 151,828** | | **eGFR ≥ 60**  **n= 1,160,165** | |
|  |  | **OR**^†^**_adj_ (95%CI**^‡^**)** | **p-value** | **OR**^†^**_adj_ (95%CI**^‡^**)** | **p-value** |  | **OR**^†^**_adj_ (95%CI**^‡^**)** | **p-value** | **OR**^†^**_adj_ (95%CI**^‡^**)** | **p-value** |
| **Age** | |  |  |  |  |  |  |  |  |  |
|  | **Less than 60** | Reference |  | Reference |  |  | Reference |  | Reference |  |
|  | ≥**60 to** <**65** | 1.04 (0.98, 1.11) | 0.2 | 0.99 (0.95, 1.02) | <0.001 |  | 1.22 (1.16, 1.27) | <0.001 | 0.97 (0.95, 0.99) | 0.002 |
|  | ≥**65 to** <**70** | 1.07 (1.01, 1.14) | 0.03 | 1.03 (1.01, 1.06) | 0.5 |  | 1.29 (1.22, 1.37) | <0.001 | 1.00 (0.97, 1.03) | 0.9 |
|  | ≥**70** | 1.09 (1.02, 1.17) | 0.01 | 1.09 (1.05, 1.14) | 0.02 |  | 1.44 (1.36, 1.53) | <0.001 | 1.04 (1.01, 1.08) | 0.004 |
| **Sex** | |  |  |  |  |  |  |  |  |  |
|  | **Man** | Reference |  | Reference |  |  | Reference |  | Reference |  |
|  | **Woman** | 0.77 (0.70, 0.85) | <0.001 | 1.01 (0.99, 1.04) | 0.3 |  | 0.81 (0.77, 0.85) | <0.001 | 1.06 (1.04, 1.08) | <0.001 |
| **Hemoglobin A1c, %** | | |  |  |  |  |  |  |  |  |
|  | **Less than 6.2** | Reference |  | Reference |  |  | Reference |  | Reference |  |
|  | ≥**6.2 to** <**6.9** | 0.93 (0.87, 1.00) | 0.05 | 0.96 (0.93, 0.99) | 0.01 |  | 1.04 (0.98, 1.11) | 0.2 | 1.01 (0.99, 1.03) | 0.4 |
|  | ≥**6.9 to** <**7.4** | 0.94 (0.86, 1.02) | 0.1 | 0.99 (0.95, 1.03) | 0.6 |  | - |  | - |  |
|  | ≥**7.4 to** <**8.4** | 1.01 (0.93, 1.10) | 0.8 | 1.11 (1.07, 1.15) | <0.001 |  | - |  | - |  |
|  | ≥**8.4** | 1.51 (1.35, 1.69) | <0.001 | 1.82 (1.75, 1.89) | <0.001 |  | - |  | - |  |
| **Body mass index, kg/m^2^** | | |  |  |  |  |  |  |  |  |
|  | **Less than 18.5** | 0.95 (0.76, 1.20) | 0.7 | 0.95 (0.88, 1.02) | 0.2 |  | 0.90 (0.82, 1.00) | 0.06 | 0.95 (0.93, 0.97) | <0.001 |
|  | ≥**18.5 to** <**25** | Reference |  | Reference |  |  | Reference |  | Reference |  |
|  | ≥**25** | 1.06 (1.02, 1.11) | 0.009 | 1.01 (0.99, 1.03) | 0.2 |  | 1.13 (1.09, 1.17) | <0.001 | 1.05 (1.04, 1.07) | <0.001 |
| **Systolic blood pressure, mmHg** | | |  |  |  |  |  |  |  |  |
|  | **Less than 120** | Reference |  | Reference |  |  | Reference |  | Reference |  |
|  | ≥**120 to** <**130** | 1.14 (1.06, 1.22) | <0.001 | 1.08 (1.05, 1.12) | <0.001 |  | 1.16 (1.11, 1.22) | 0.03 | 1.06 (1.05, 1.07) | <0.001 |
|  | ≥**130 to** <**140** | 1.37 (1.25, 1.50) | <0.001 | 1.18 (1.15, 1.21) | <0.001 |  | 1.30 (1.23, 1.36) | <0.001 | 1.11 (1.10, 1.13) | 0.002 |
|  | ≥**140 to** <**150** | 1.54 (1.36, 1.73) | <0.001 | 1.24 (1.17, 1.31) | <0.001 |  | 1.42 (1.35, 1.50) | <0.001 | 1.19 (1.16, 1.21) | <0.001 |
|  | ≥**150 to** <**160** | 1.60 (1.45, 1.76) | <0.001 | 1.34 (1.29, 1.39) | <0.001 |  | 1.46 (1.32, 1.61) | <0.001 | 1.24 (1.21, 1.28) | <0.001 |
|  | ≥**160** | 2.09 (1.78, 2.45) | <0.001 | 1.45 (1.40, 1.50) | <0.001 |  | 1.93 (1.78, 2.09) | <0.001 | 1.33 (1.29, 1.37) | <0.001 |
| **Proteinuria** | |  |  |  |  |  |  |  |  |  |
|  | **-** | Reference |  | Reference |  |  | Reference |  | Reference |  |
|  | **±** | 1.21 (1.11, 1.31) | <0.001 | 0.99 (0.94, 1.03) | 0.5 |  | 1.06 (1.01, 1.12) | <0.001 | 0.88 (0.86, 0.91) | <0.001 |
|  | **1+** | 1.75 (1.61, 1.91) | <0.001 | 1.10 (1.06, 1.15) | <0.001 |  | 1.76 (1.63, 1.90) | <0.001 | 0.92 (0.88, 0.97) | <0.001 |
|  | **2+** | 3.66 (3.39, 3.96) | <0.001 | 1.56 (1.47, 1.65) | <0.001 |  | 3.73 (3.41, 4.08) | <0.001 | 1.23 (1.15, 1.30) | <0.001 |
|  | **3+** | 7.65 (6.77, 8.66) | <0.001 | 1.95 (1.75, 2.17) | <0.001 |  | 6.73 (5.96, 7.60) | <0.001 | 1.39 (1.21, 1.58) | <0.001 |
| **LDL-C**^§^**, mg/dl** | | |  |  |  |  |  |  |  |  |
|  | **Less than 100** | Reference |  | Reference |  |  | Reference |  | Reference |  |
|  | ≥**100 to** <**120** | 0.90 (0.82, 0.97) | <0.001 | 0.90 (0.87, 0.93) | <0.001 |  | 0.85 (0.81, 0.89) | <0.001 | 0.90 (0.89, 0.91) | <0.001 |
|  | ≥**120** | 0.80 (0.76, 0.85) | <0.001 | 0.82 (0.80, 0.85) | <0.001 |  | 0.76 (0.73, 0.79) | <0.001 | 0.82 (0.81, 0.83) | <0.001 |
| **Hemoglobin, g/dL** | | |  |  |  |  |  |  |  |  |
|  | **Less than 9** | 1.38 (0.48, 3.95) | <0.001 | 1.54 (1.21, 1.98) | <0.001 |  | 1.77 (1.17, 2.69) | 0.007 | 1.56 (1.45, 1.69) | <0.001 |
|  | ≥**9 to** <**11** | 3.21 (2.78, 3.71) | <0.001 | 1.30 (1.15, 1.47) | <0.001 |  | 2.47 (2.08, 2.93) | <0.001 | 1.19 (1.15, 1.22) | <0.001 |
|  | ≥**11 to** <**13** | 2.04 (1.89, 2.21) | <0.001 | 1.30 (1.23, 1.38) | <0.001 |  | 1.69 (1.59, 1.80) | <0.001 | 1.21 (1.18, 1.24) | <0.001 |
|  | ≥**13** | Reference |  | Reference |  |  | Reference |  | Reference |  |
| **Variance** | | < 0.001 | | 0.034 (0.024, 0.048) | |  | 0.025 (0.014, 0.046) | | 0.079 (0.05, 0.097) | |
| **Num. of insurers^**^** | | 1,257 | | 1,706 | |  | 1,739 | | 2,139 | |

Rapid decline, %slope in estimated glomerular filtration rate is over -10%, non-rapid decline, %slope in estimated glomerular filtration rate is less than -10%.

*eGFR, estimated glomerular filtration rate.

†OR_adj_, odds ratio adjusted for age, Sex, hemoglobinA1c, body mass index, systolic blood pressure, proteinuria, low-density lipoprotein cholesterol, hemoglobin, current smoking, and alcohol consumption.

‡95%CI, 95% confidence intervals.

§LDL-C, low-density lipoprotein cholesterol.

¶Variance, variance in insurer level.

**Num. of insurers, number of insurers.

**Supplemental Table 3. Odds ratio of rapid decline in renal function according to subgroups (lipid-lowering drugs).**

|  |  | **Participants with diabetes** | | | |  | **Participants without diabetes** | | | |
| --- | --- | --- | --- | --- | --- | --- | --- | --- | --- | --- |
|  |  | **With lipid-lowering drugs**  **n = 105,292** | | **Without lipid-lowering drugs**  **n = 192,661** | |  | **With lipid-lowering drugs**  **n = 195,303** | | **Without lipid-lowering drugs**  **n = 1,116,640** | |
|  |  | **OR**^†^**_adj_ (95%CI**^‡^**)** | **p-value** | **OR**^†^**_adj_ (95%CI**^‡^**)** | **p-value** |  | **OR**^†^**_adj_ (95%CI**^‡^**)** | **p-value** | **OR**^†^**_adj_ (95%CI**^‡^**)** | **p-value** |
| **Age** | |  |  |  |  |  |  |  |  |  |
|  | **Less than 60** | Reference |  | Reference |  |  | Reference |  | Reference |  |
|  | ≥**60 to** <**65** | 0.97 (0.94,1.00) | 0.09 | 0.96 (0.92,1.00) | 0.04 |  | 0.96 (0.93,1.00) | 0.03 | 0.93 (0.91,0.95) | <0.001 |
|  | ≥**65 to** <**70** | 1.00 (0.96,1.04) | 0.9 | 0.98 (0.95,1.01) | 0.2 |  | 0.98 (0.94,1.02) | 0.3 | 0.94 (0.91,0.97) | <0.001 |
|  | ≥**70** | 1.02 (0.96,1.09) | 0.5 | 1.02 (0.98,1.07) | 0.4 |  | 1.01 (0.97,1.05) | 0.6 | 0.97 (0.94,1.00) | 0.09 |
| **Sex** | |  |  |  |  |  |  |  |  |  |
|  | **Man** | Reference |  | Reference |  |  | Reference |  | Reference |  |
|  | **Woman** | 0.97 (0.92,1.01) | 0.2 | 1.03 (1.00,1.07) | 0.04 |  | 1.06 (1.02,1.11) | 0.004 | 1.06 (1.04,1.08) | <0.001 |
| **Hemoglobin A1c, %** | | |  |  |  |  |  |  |  |  |
|  | **Less than 6.2** | Reference |  | Reference |  |  | Reference |  | Reference |  |
|  | ≥**6.2 to** <**6.9** | 0.92 (0.88,0.97) | 0.001 | 0.98 (0.95,1.01) | 0.2 |  | 1.03 (1.00,1.06) | 0.1 | 1.01 (0.99,1.03) | 0.4 |
|  | ≥**6.9 to** <**7.4** | 0.93 (0.89,0.98) | 0.005 | 1.02 (0.98,1.06) | 0.3 |  |  |  |  |  |
|  | ≥**7.4 to** <**8.4** | 1.05 (1.00,1.11) | 0.05 | 1.14 (1.08,1.20) | <0.001 |  |  |  |  |  |
|  | ≥**8.4** | 1.49 (1.41,1.58) | <0.0001 | 1.95 (1.84,2.07) | <0.001 |  |  |  |  |  |
| **Body mass index, kg/m^2^** | | |  |  |  |  |  |  |  |  |
|  | **Less than 18.5** | 0.96 (0.81,1.14) | 0.6 | 0.94 (0.87,1.01) | 0.1 |  | 0.98 (0.91,1.06) | 0.7 | 0.95 (0.93,0.98) | <0.001 |
|  | ≥**18.5 to** <**25** | Reference |  | Reference |  |  | Reference |  | Reference |  |
|  | ≥**25** | 1.03 (1.00,1.07) | 0.09 | 0.99 (0.97,1.01) | 0.4 |  | 1.04 (1.02,1.07) | 0.002 | 1.04 (1.03,1.06) | <0.001 |
| **Systolic blood pressure, mmHg** | | |  |  |  |  |  |  |  |  |
|  | **Less than 120** | Reference |  | Reference |  |  | Reference |  | Reference |  |
|  | ≥**120 to** <**130** | 1.12 (1.07,1.16) | <0.001 | 1.09 (1.04,1.14) | <0.001 |  | 1.09 (1.06,1.13) | <0.001 | 1.07 (1.05,1.08) | <0.001 |
|  | ≥**130 to** <**140** | 1.28 (1.24,1.32) | <0.001 | 1.17 (1.12,1.22) | <0.001 |  | 1.18 (1.15,1.22) | <0.001 | 1.12 (1.10,1.14) | <0.001 |
|  | ≥**140 to** <**150** | 1.30 (1.21,1.40) | <0.001 | 1.28 (1.19,1.36) | <0.001 |  | 1.28 (1.24,1.33) | <0.001 | 1.19 (1.17,1.22) | <0.001 |
|  | ≥**150 to** <**160** | 1.43 (1.34,1.52) | <0.001 | 1.36 (1.29,1.42) | <0.001 |  | 1.28 (1.21,1.35) | <0.001 | 1.26 (1.22,1.30) | <0.001 |
|  | ≥**160** | 1.67 (1.54,1.81) | <0.001 | 1.49 (1.40,1.58) | <0.001 |  | 1.48 (1.38,1.60) | <0.001 | 1.36 (1.32,1.40) | <0.001 |
| **Proteinuria** | |  |  |  |  |  |  |  |  |  |
|  | **-** | Reference |  | Reference |  |  | Reference |  | Reference |  |
|  | **±** | 1.04 (0.97,1.11) | 0.3 | 0.97 (0.93,1.02) | 0.2 |  | 0.95 (0.91,1.00) | 0.05 | 0.88 (0.85,0.90) | <0.001 |
|  | **1+** | 1.18 (1.12,1.24) | <0.001 | 1.13 (1.08,1.18) | <0.001 |  | 1.12 (1.05,1.19) | 0.001 | 0.93 (0.89,0.98) | 0.003 |
|  | **2+** | 2.04 (1.90,2.19) | <0.001 | 1.76 (1.65,1.88) | <0.001 |  | 1.96 (1.81,2.13) | <0.001 | 1.39 (1.32,1.47) | <0.001 |
|  | **3+** | 3.39 (3.04,3.78) | <0.000 | 3.39 (3.05,3.76) | <0.001 |  | 3.36 (2.84,3.97) | <0.001 | 2.04 (1.82,2.28) | <0.001 |
| **LDL-C**^§^**, mg/dl** | | |  |  |  |  |  |  |  |  |
|  | **Less than 100** | Reference |  | Reference |  |  | Reference |  | Reference |  |
|  | ≥**100 to** <**120** | 0.89 (0.86,0.93) | <0.001 | 0.89 (0.87,0.92) | <0.001 |  | 0.89 (0.86,0.92) | <0.001 | 0.90 (0.89,0.91) | <0.001 |
|  | ≥**120** | 0.87 (0.83,0.90) | <0.001 | 0.78 (0.76,0.81) | <0.001 |  | 0.83 (0.81,0.86) | <0.001 | 0.80 (0.79,0.81) | <0.001 |
| **Hemoglobin, g/dL** | | |  |  |  |  |  |  |  |  |
|  | **Less than 9** | 2.23 (1.36,3.65) | 0.001 | 1.36 (0.99,1.88) | 0.06 |  | 1.23 (0.70,2.16) | 0.5 | 1.62 (1.51,1.75) | <0.001 |
|  | ≥**9 to** <**11** | 2.11 (1.79,2.49) | <0.001 | 1.54 (1.37,1.72) | <0.001 |  | 1.56 (1.35,1.82) | <0.001 | 1.24 (1.19,1.29) | <0.001 |
|  | ≥**11 to** <**13** | 1.43 (1.30,1.58) | <0.001 | 1.40 (1.33,1.47) | <0.001 |  | 1.24 (1.20,1.29) | <0.001 | 1.24 (1.21,1.27) | <0.001 |
|  | ≥**13** | Reference |  | Reference |  |  | Reference |  | Reference |  |
| **Variance** | | 0.36 (0.019,0.051) | | 0.029 (0.20,0.044) | |  | 0.051 (0.036, 0.074) | | 0.078 (0.064, 0.096) | |
| **Num. of insurers^**^** | | 1,486 | | 1,644 | |  | 1,746 | | 2,133 | |

Rapid decline, %slope in estimated glomerular filtration rate is over -10%, non-rapid decline, %slope in estimated glomerular filtration rate is less than -10%.

*eGFR, estimated glomerular filtration rate.

†OR_adj_, odds ratio adjusted for age, Sex, hemoglobinA1c, body mass index, systolic blood pressure, proteinuria, low-density lipoprotein cholesterol, hemoglobin, current smoking, and alcohol consumption.

‡95%CI, 95% confidence intervals.

§LDL-C, low-density lipoprotein cholesterol.

¶Variance, variance in insurer level.

**Num. of insurers, number of insurers.

**Supplemental Table 4. Odds ratio of rapid decline in renal function according to subgroups (anti-hypertensive).**

|  |  | **Participants with diabetes** | | | |  | **Participants without diabetes** | | | |
| --- | --- | --- | --- | --- | --- | --- | --- | --- | --- | --- |
|  |  | **With anti-hypertensive drugs**  **n = 138,462** | | **Without anti-hypertensive drugs**  **n = 159,498** | |  | **With anti-hypertensive drugs**  **n = 272,274** | | **Without anti-hypertensive drugs**  **n = 1,039,677** | |
|  |  | **OR**^†^**_adj_ (95%CI**^‡^**)** | **p-value** | **OR**^†^**_adj_ (95%CI**^‡^**)** | **p-value** |  | **OR**^†^**_adj_ (95%CI**^‡^**)** | **p-value** | **OR**^†^**_adj_ (95%CI**^‡^**)** | **p-value** |
| **Age** | |  |  |  |  |  |  |  |  |  |
|  | **Less than 60** | Reference |  | Reference |  |  | Reference |  | Reference |  |
|  | ≥**60 to** <**65** | 0.97 (0.93,1.01) | 0.1 | 0.92 (0.89,0.95) | <0.001 |  | 0.99 (0.96,1.02) | 0.4 | 0.89 (0.87,0.91) | <0.001 |
|  | ≥**65 to** <**70** | 0.98 (0.95,1.02) | 0.4 | 0.93 (0.89,0.98) | 0.002 |  | 0.99 (0.96,1.02) | 0.6 | 0.89 (0.87,0.92) | <0.001 |
|  | ≥**70** | 1.01 (0.97,1.06) | 0.6 | 0.95 (0.90,1.00) | 0.05 |  | 1.03 (0.99,1.06) | 0.1 | 0.91 (0.88,0.94) | <0.001 |
| **Sex** | |  |  |  |  |  |  |  |  |  |
|  | **Man** | Reference |  | Reference |  |  | Reference |  | Reference |  |
|  | **Woman** | 0.98 (0.94,1.02) | 0.3 | 1.03 (1.00,1.07) | 0.05 |  | 1.05 (1.02,1.08) | 0.002 | 1.06 (1.04,1.08) | <0.001 |
| **Hemoglobin A1c, %** | | |  |  |  |  |  |  |  |  |
|  | **Less than 6.2** | Reference |  | Reference |  |  | Reference |  | Reference |  |
|  | ≥**6.2 to** <**6.9** | 0.96 (0.92,0.99) | 0.02 | 0.97 (0.93,1.02) | 0.3 |  | 0.99 (0.95,1.03) | 0.7 | 1.01 (0.98,1.04) | 0.4 |
|  | ≥**6.9 to** <**7.4** | 0.97 (0.92,1.01) | 0.1 | 1.04 (0.97,1.10) | 0.3 |  |  |  |  |  |
|  | ≥**7.4 to** <**8.4** | 1.06 (1.01,1.12) | 0.02 | 1.18 (1.13,1.23) | <0.001 |  |  |  |  |  |
|  | ≥**8.4** | 1.52 (1.44,1.61) | <0.001 | 2.11 (2.02,2.21) | <0.001 |  |  |  |  |  |
| **Body mass index, kg/m^2^** | | |  |  |  |  |  |  |  |  |
|  | **Less than 18.5** | 0.97 (0.85,1.10) | 0.6 | 0.99 (0.91,1.07) | 0.8 |  | 1.04 (0.98,1.11) | 0.2 | 0.96 (0.94,0.98) | 0.001 |
|  | ≥**18.5 to** <**25** | Reference |  | Reference |  |  | Reference |  | Reference |  |
|  | ≥**25** | 0.99 (0.96,1.02) | 0.5 | 0.98 (0.95,1.01) | 0.1 |  | 1.01 (0.99,1.03) | 0.4 | 1.04 (1.02,1.05) | <0.001 |
| **Systolic blood pressure, mmHg** | | |  |  |  |  |  |  |  |  |
|  | **Less than 120** | Reference |  | Reference |  |  | Reference |  | Reference |  |
|  | ≥**120 to** <**130** | 1.16 (1.11,1.21) | <0.001 | 1.05 (1.01,1.09) | 0.005 |  | 1.09 (1.07,1.12) | <0.001 | 1.06 (1.05,1.07) | <0.001 |
|  | ≥**130 to** <**140** | 1.3 (1.23,1.37) | <0.001 | 1.12 (1.09,1.16) | <0.001 |  | 1.17 (1.13,1.20) | <0.001 | 1.11 (1.09,1.13) | <0.001 |
|  | ≥**140 to** <**150** | 1.36 (1.28,1.45) | <0.001 | 1.21 (1.12,1.30) | <0.001 |  | 1.27 (1.23,1.31) | <0.001 | 1.17 (1.14,1.19) | <0.001 |
|  | ≥**150 to** <**160** | 1.46 (1.39,1.54) | <0.001 | 1.31 (1.25,1.37) | <0.001 |  | 1.28 (1.22,1.34) | <0.001 | 1.25 (1.21,1.29) | <0.001 |
|  | ≥**160** | 1.65 (1.56,1.74) | <0.001 | 1.47 (1.38,1.56) | <0.001 |  | 1.41 (1.34,1.47) | <0.001 | 1.36 (1.32,1.41) | <0.001 |
| **Proteinuria** | |  |  |  |  |  |  |  |  |  |
|  | **-** | Reference |  | Reference |  |  | Reference |  | Reference |  |
|  | **±** | 1.01 (0.97,1.06) | 0.6 | 0.97 (0.92,1.02) | 0.3 |  | 0.96 (0.92,0.99) | 0.02 | 0.87 (0.84,0.89) | <0.001 |
|  | **1+** | 1.21 (1.15,1.27) | <0.001 | 1.04 (0.98,1.10) | 0.2 |  | 1.15 (1.10,1.21) | <0.001 | 0.85 (0.80,0.90) | <0.001 |
|  | **2+** | 1.98 (1.87,2.09) | <0.001 | 1.55 (1.45,1.66) | <0.001 |  | 1.85 (1.69,2.02) | <0.001 | 1.17 (1.07,1.29) |  |
|  | **3+** | 3.61 (3.23,4.05) | <0.001 | 2.49 (2.20,2.83) | <0.001 |  | 3.09 (2.77,3.46) | <0.001 | 1.45 (1.27,1.67) | <0.001 |
| **LDL-C**^§^**, mg/dl** | | |  |  |  |  |  |  |  |  |
|  | **Less than 100** | Reference |  | Reference |  |  | Reference |  | Reference |  |
|  | ≥**100 to** <**120** | 0.91 (0.89,0.94) | <0.001 | 0.90 (0.87,0.94) | <0.001 |  | 0.92 (0.89,0.94) | <0.001 | 0.90 (0.89,0.91) | <0.001 |
|  | ≥**120** | 0.84 (0.81,0.87) | <0.001 | 0.83 (0.81,0.86) | <0.001 |  | 0.84 (0.82,0.86) | <0.001 | 0.81 (0.80,0.82) | <0.001 |
| **Hemoglobin, g/dL** | | |  |  |  |  |  |  |  |  |
|  | **Less than 9** | 1.56 (0.98,2.48) | 0.06 | 1.53 (1.16,2.01) | 0.003 |  | 1.73 (1.19,2.52) | 0.004 | 1.63 (1.52,1.74) | <0.001 |
|  | ≥**9 to** <**11** | 2.13 (1.87,2.41) | <0.001 | 1.34 (1.16,1.55) | <0.001 |  | 1.47 (1.34,1.61) | <0.001 | 1.24 (1.20,1.29) | <0.001 |
|  | ≥**11 to** <**13** | 1.47 (1.39,1.56) | <0.001 | 1.31 (1.21,1.41) | <0.001 |  | 1.25 (1.20,1.29) | <0.001 | 1.24 (1.21,1.27) | <0.001 |
|  | ≥**13** | Reference |  | Reference |  |  | Reference |  | Reference |  |
| **Variance** | | 0.019 (0.012, 0.029) | | 0.046 (0.032, 0.068) | |  | 0.046 (0.032, 0.066) | | 0.081 (0.065, 0.099) | |
| **Num. of insurers^**^** | | 1,556 | | 1,571 | |  | 1,798 | | 2,118 | |

Rapid decline, %slope in estimated glomerular filtration rate is over -10%, non-rapid decline, %slope in estimated glomerular filtration rate is less than -10%.

*eGFR, estimated glomerular filtration rate.

†OR_adj_, odds ratio adjusted for age, Sex, hemoglobinA1c, body mass index, systolic blood pressure, proteinuria, low-density lipoprotein cholesterol, hemoglobin, current smoking, and alcohol consumption.

‡95%CI, 95% confidence intervals.

§LDL-C, low-density lipoprotein cholesterol.

¶Variance, variance in insurer level.

**Num. of insurers, number of insurers.

**Supplemental Table 5. Odds ratio associated with slope of eGFR (10 ml/min/1.73 m2/year or greater decline).**

|  |  | **Participants with diabetes**  **n = 297,972** | | | |  | **Participants without diabetes**  **n = 1,311,501** | | | |
| --- | --- | --- | --- | --- | --- | --- | --- | --- | --- | --- |
|  |  | **Crude** | | **Adjusted** | |  | **Crude** | | **Adjusted** | |
|  |  | **OR^*^ (95%CI**^†^**)** | **p-value** | **OR^*^ (95%CI**^†^**)** | **p-value** |  | **OR^*^ (95%CI**^†^**)** | **p-value** | **OR^*^ (95%CI**^†^**)** | **p-value** |
| **Baseline eGFR**^‡^ | |  |  |  |  |  |  |  |  |  |
|  | **Or more 90** | 4.18 (4.12, 4.25) | <0.001 | 3.95 (3.86,4.04) | <0.001 |  | 5.17 (5.11, 5.23) | <0.001 | 5.15 (5.06,5.24) | <0.001 |
|  | **≥60 to <90** | Reference |  | Reference |  |  | Reference |  | Reference |  |
|  | **≥45 to <60** | 0.36 (0.34, 0.38) | <0.001 | 0.34 (0.31,0.37) | <0.001 |  | 0.19 (0.18, 0.20) | <0.001 | 0.18 (0.17,0.19) | <0.001 |
|  | **≥30 to <45** | 0.50 (0.42, 0.58) | <0.001 | 0.38 (0.35,0.42) | <0.001 |  | 0.24 (0.22, 0.27) | <0.001 | 0.22 (0.19,0.25) | <0.001 |
|  | **≥15 to <30** | 0.94 (0.72, 1.22) | 0.6 | 0.42 (0.34,0.51) | <0.001 |  | 0.36 (0.28, 0.46) | <0.001 | 0.23 (0.17,0.32) | <0.001 |
|  | **Less than 15** | 0.026 (0.011, 0.062) | <0.001 | 0.02 (0.00,0.07) | <0.001 |  | - |  | - |  |
| **Age** | |  |  |  |  |  |  |  |  |  |
|  | **Less than 60** | Reference |  | Reference |  |  | Reference |  | Reference |  |
|  | ≥**60 to** <**65** | 0.76 (0.74,0.78) | <0.001 | 1.04 (1.00,1.07) | 0.06 |  | 0.78 (0.77,0.79) | <0.001 | 0.97 (0.95,0.99) | 0.01 |
|  | ≥**65 to** <**70** | 0.70 (0.68,0.73) | <0.001 | 1.07 (1.03,1.11) | <0.001 |  | 0.76 (0.74,0.78) | <0.001 | 1.01 (0.98,1.04) | 0.5 |
|  | ≥**70** | 0.66 (0.63,0.68) | <0.001 | 1.11 (1.06,1.18) | <0.001 |  | 0.75 (0.73,0.76) | <0.001 | 1.09 (1.05,1.13) | <0.001 |
| **Sex** | |  |  |  |  |  |  |  |  |  |
|  | **Man** | Reference |  | Reference |  |  | Reference |  | Reference |  |
|  | **Woman** | 1.09 (1.07,1.11) | <0.001 | 0.98 (0.95,1.01) | 0.2 |  | 1.16 (1.15,1.18) | <0.001 | 1.07 (1.04,1.09) | <0.001 |
| Hemoglobin A1c, % | | |  |  |  |  |  |  |  |  |
|  | **Less than 6.2** | Reference |  | Reference |  |  | Reference |  | Reference |  |
|  | ≥**6.2 to** <**6.9** | 0.94 (0.92,0.96) | <0.001 | 0.92 (0.89,0.95) | <0.001 |  | 1.02 (1.00,1.05) | 0.1 | 1.03 (1.00,1.05) | 0.03 |
|  | ≥**6.9 to** <**7.4** | 1.04 (1.01,1.06) | 0.005 | 0.95 (0.92,0.99) | 0.02 |  | - | - | - | - |
|  | ≥**7.4 to** <**8.4** | 1.27 (1.24,1.30) | <0.001 | 1.03 (0.99,1.07) | 0.1 |  | - | - | - | - |
|  | ≥**8.4** | 2.49 (2.42,2.55) | <0.001 | 1.65 (1.58,1.72) | <0.001 |  | - | - | - | - |
| Body mass index, kg/m^2^ | | |  |  |  |  |  |  |  |  |
|  | **Less than 18.5** | 1.13 (1.06,1.21) | <0.001 | 0.91 (0.84,0.99) | 0.04 |  | 0.08 (0.08,0.08) | <0.001 | 0.94 (0.92,0.97) | <0.001 |
|  | ≥**18.5 to** <**25** | Reference |  | Reference |  |  | Reference |  | Reference |  |
|  | ≥**25** | 1.06 (1.04,1.08) | <0.001 | 1.01 (0.99,1.04) | 0.3 |  | 0.98 (0.97,1.00) | 0.05 | 1.07 (1.05,1.08) | <0.001 |
| Systolic blood pressure, mmHg | | |  |  |  |  |  |  |  |  |
|  | **Less than 120** | Reference |  | Reference |  |  | Reference |  | Reference |  |
|  | ≥**120 to** <**130** | 1.10 (1.08,1.12) | <0.001 | 1.09 (1.06,1.11) | <0.001 |  | 1.02 (1.01,1.03) | 0.006 | 1.04 (1.03,1.06) | <0.001 |
|  | ≥**130 to** <**140** | 1.20 (1.17,1.24) | <0.001 | 1.19 (1.14,1.24) | <0.001 |  | 1.06 (1.04,1.07) | <0.001 | 1.10 (1.08,1.12) | <0.001 |
|  | ≥**140 to** <**150** | 1.29 (1.24,1.35) | <0.001 | 1.25 (1.19,1.31) | <0.001 |  | 1.12 (1.09,1.15) | <0.001 | 1.17 (1.14,1.19) | <0.001 |
|  | ≥**150 to** <**160** | 1.44 (1.39,1.48) | <0.001 | 1.37 (1.32,1.42) | <0.001 |  | 1.19 (1.16,1.23) | <0.001 | 1.24 (1.20,1.28) | <0.001 |
|  | ≥**160** | 1.67 (1.61,1.72) | <0.001 | 1.54 (1.47,1.60) | <0.001 |  | 1.29 (1.24,1.33) | <0.001 | 1.32 (1.27,1.37) | <0.001 |
| **Proteinuria** | |  |  |  |  |  |  |  |  |  |
|  | **-** | Reference |  | Reference |  |  | Reference |  | Reference |  |
|  | **±** | 1.04 (1.01,1.07) | 0.008 | 0.99 (0.95,1.03) | 0.6 |  | 0.92 (0.90,0.94) | <0.001 | 0.92 (0.89,0.95) | <0.001 |
|  | **1+** | 1.22 (1.19,1.25) | <0.001 | 1.15 (1.11,1.19) | <0.001 |  | 0.91 (0.89,0.94) | <0.001 | 0.97 (0.91,1.03) | 0.3 |
|  | **2+** | 1.61 (1.54,1.68) | <0.001 | 1.86 (1.75,1.99) | <0.001 |  | 1.07 (1.03,1.12) | 0.001 | 1.53 (1.41,1.67) | <0.001 |
|  | **3+** | 2.23 (1.99,2.49) | <0.001 | 3.48 (3.05,3.97) | <0.001 |  | 1.34 (1.19,1.50) | <0.001 | 2.49 (2.07,3.00) | <0.001 |
| LDL-C^§^, mg/dl | | |  |  |  |  |  |  |  |  |
|  | **Less than 100** | Reference |  | Reference |  |  | Reference |  | Reference |  |
|  | ≥**100 to** <**120** | 0.90 (0.89,0.92) | <0.001 | 0.89 (0.87,0.92) | <0.001 |  | 0.87 (0.86,0.88) | <0.001 | 0.89 (0.88,0.90) | <0.001 |
|  | ≥**120** | 0.89 (0.88,0.91) | <0.001 | 0.82 (0.80,0.84) | <0.001 |  | 0.76 (0.75,0.77) | <0.001 | 0.80 (0.79,0.82) | <0.001 |
| Hemoglobin, g/dL | | |  |  |  |  |  |  |  |  |
|  | **Less than 9** | 1.55 (1.30,1.84) | <0.001 | 1.37 (1.05,1.80) | 0.02 |  | 2.37 (2.23,2.52) | <0.001 | 1.38 (1.26,1.52) | <0.001 |
|  | ≥**9 to** <**11** | 1.24 (1.14,1.35) | <0.001 | 1.44 (1.29,1.61) | <0.001 |  | 1.62 (1.57,1.67) | <0.001 | 1.12 (1.08,1.16) | <0.001 |
|  | ≥**11 to** <**13** | 1.14 (1.09,1.20) | <0.001 | 1.34 (1.26,1.42) | <0.001 |  | 1.29 (1.25,1.32) | <0.001 | 1.20 (1.17,1.23) | <0.001 |
|  | ≥**13** | Reference |  | Reference | <0.001 |  | Reference |  | Reference |  |
|  | Variance in insurer level | |  | 0.032 (0.021, 0.048) | |  |  |  | 0.087 (0.071, 0.11) | |
|  | Number of insurers | |  | 1,754 | |  |  |  | 2,176 | |

Rapid decline, slope in estimated glomerular filtration rate is over -10 ml/min/1.73 m2/year, non-rapid decline, slope in estimated glomerular filtration rate is less than -10 ml/min/1.73 m2/year.

*OR, odds ratio, adjusted for current smoking, quantity of drinking alcohol.

†95%CI, 95% confidence intervals.

‡eGFR, estimated glomerular filtration rate (mL/min/1.73m^2^).

§LDL-C, low-density lipoprotein cholesterol.

**Supplemental Figure. Subgroup Analysis for Rapid Renal Decline in Blood Pressure.**

**Supplemental Table 6.** Relationship between hemoglobin level at baseline and renal function.

|  | **mean (95%CI^*^)** | **Adjusted marginal**  **mean^†^ (95%CI)** | **p-value^‡^** | **p-value^§^** |
| --- | --- | --- | --- | --- |
| **Hemoglobin, g/dL** | |  |  |  |
| **Less than 9** | **80.56 (78.6, 82.52)** | **79.24 (77.45, 81.03)** | **<0.001** | **<0.001** |
| **≥9 to <11** | **67.91 (67.05, 68.76)** | **68.86 (68.2, 69.52)** |  | **<0.001** |
| **≥11 to <13** | **71.14 (70.93, 71.35)** | **71.58 (71.36, 71.81)** |  | **<0.001** |
| **≥13** | **76.67 (76.62, 76.72)** | **76.64 (76.58, 76.7)** |  | **Reference** |

**^*^95% CI, 95% confidence intervals.**

**^†^Adjusted for age, sex, hemoglobin A1c, body mass index, systolic blood pressure, proteinuria, low-density lipoprotein cholesterol, current smoking, and alcohol consumption.**

**^‡^p-value for analysis of variance.**

**^§^p-value for the Dunnet test.**
